# Thermophysical and Rheological Characteristics of CO_2_ Hydrate Slurries for Cold Thermal Energy Storage Applications and Engineering Perspectives

**DOI:** 10.3390/ma19071434

**Published:** 2026-04-03

**Authors:** Sai Bhargav Annavajjala, Noah Van Dam, Jan Kosny

**Affiliations:** Department of Mechanical and Industrial Engineering, University of Massachusetts Lowell, Lowell, MA 01854, USA; saibhargav_annavajjala@student.uml.edu (S.B.A.); noah_vandam@uml.edu (N.V.)

**Keywords:** CO_2_ hydrate slurry, cold thermal energy storage (CTES), hydrate nucleation kinetics, rheological properties, surfactants and nanoparticles

## Abstract

Carbon dioxide (CO_2_) hydrate slurries have emerged as promising candidates for cold thermal energy storage (CTES) and refrigeration systems due to their high latent heat, controllable flow behavior, and environmentally friendly nature. These slurries are formed by dispersing solid CO_2_ hydrate particles in a liquid phase, forming a multiphase system with tunable thermophysical and rheological properties. The performance of these slurries is dependent on nucleation kinetics, particle sizes and their distribution, solid content, and thermal transport characteristics under flow conditions. This review paper gives an assessment of CO_2_ hydrate slurries from a thermofluid’ perspective by focusing on key aspects such as hydrate nucleation mechanisms, viscosity behavior, shear response, thermal conductivity, convective heat transfer, and slurry stability. Particular attention is given to the role of surfactants and nanoparticle additives that enhance hydrate formation and improve slurry performance. The addition of nanofluids is discussed both in terms of their effect on thermal properties as well as in flow stability.

## 1. Introduction

Over the past two decades, there has been a growing emphasis on developing sustainable and energy-efficient solutions for refrigeration, air conditioning, and thermal management systems. Cooling demand is increasing rapidly due to urbanization, data-center expansion, and electrification, placing significant pressure on conventional refrigeration systems that rely on high-global-warming-potential refrigerants. Cold thermal energy storage (CTES) technologies have emerged as a viable pathway to decouple cooling demand from energy supply while improving system efficiency and reducing emissions. This transition is driven not only by the global increase in cooling demand but also by the urgent need to reduce greenhouse gas emissions, including the elimination of high-global-warming-potential (GWP) refrigerants, which are widely used in space air conditioning. In this context, gas hydrate-based CTES systems have emerged as an innovative solution by having high energy density, environmental safety, and thermal controllability. Among various gas hydrates, CO_2_ hydrates are particularly gaining attention due to their high energy storage density, wide availability at low cost, and flame retardancy of CO_2_, as well as its favorable hydrate formation characteristics under moderate pressure and temperature conditions [1,2].

Phase change material (PCM) slurries and ice slurries are both well-known and widely considered as effective thermal carriers designed to enhance the energy transport capabilities of cooling systems. These slurries depend on the latent heat of suspended phase change materials to deliver significantly higher thermal energy per unit mass or volume compared to conventional thermo-fluids like water, water mixtures with antifreeze or glycol, or brines [3]. Typically, PCM slurries consist of microencapsulated or finely dispersed phase change materials, such as paraffin waxes or salt hydrates, suspended in a carrier fluid. The key advantage of these slurries is their ability to utilize both sensible and latent heat during the transport process. At PCM concentrations of 10–30 wt% [4], the thermal energy carried by these slurries can be approximately two to three times higher than that of water [5,6].

Because the heat is absorbed or released at nearly constant temperature during the phase change process, the outlet temperature of the fluid in heat exchangers using PCM slurries remains stable, in turn, improving thermal control and overall system efficiency. The elevated energy density of PCM slurries could help reduce the required flow rate by up to 50%. This contributes to lowering the pumping power and allows for the possible usage of smaller tubing diameters without compromising the cooling capacity. Compared to conventional thermo-fluids, these slurries also exhibit predictable pressure drops, in laminar and transitional flow regimes that make them suitable for conventional pumping and heat exchanger systems [7].

Despite many thermal benefits, PCM slurries face challenges such as phase separation, sedimentation, or the development of a yield stress at higher solid fractions. These issues complicate a long-term and continues operation. Also, refrigeration systems using PCM slurries are usually more complex with added continuous agitation or re-suspension mechanisms. In contrast, ice slurry systems address several of these shortcomings while providing comparable or even superior thermal transport benefits. With a latent heat of fusion of approximately 334 Kj·kg^−1^, ice particles contribute significantly to the energy density of slurry, and this represents higher energy density than for many organic PCMs in terms of cooling capacity per unit mass. When ice is suspended in water, the ice particles contribute to the enhanced convective heat transfer. These heat transfer coefficients can be even 2–3 times higher than those observed in conventional single-phase coolants. Ice slurries can be circulated efficiently through standard piping systems without major design changes. At low to moderate ice fractions below 20%, they behave nearly as Newtonian fluids. This can lower pumping energy by up to 30%, when compared to single-phase fluids under equivalent cooling loads [8].

Ice slurries maintain their flowability even at relatively high solid fractions (up to 25%) when particle sizes are kept fine and uniformly distributed. This provides consistent heat delivery without the need for extra agitation systems. Their compatibility with existing HVAC and refrigeration technologies, including district cooling infrastructure, makes them feasible for large-scale applications and long-distance transport by mitigating the pressure loss and thermal stratification would otherwise be major concerns. However, ice slurry systems require refrigeration equipment that is more complicated and costlier than conventional chilled-water or ice block systems. The production of ice slurry typically involves the usage of advanced techniques such as supercooling devices. These units require precise control of nucleation and crystal growth to prevent blockages and maintain a stable ice fraction, increasing both design and operational complexity. The two-phase nature of ice slurry flow leads to elevated viscosity and higher-pressure drops, and that is why it requires stronger pumps and reinforced piping networks. The presence of ice particles also accelerates wear in valves, pumps, and heat exchangers. So, all of this is further raising equipment and maintenance costs. Motivated by these developments and technology advantages, especially the improved heat transfer rates, reduced pumping energy, and system adaptability, CO_2_ hydrate technologies have increasingly shifted toward slurry-based formulations. By suspending CO_2_ hydrate particles in a liquid medium, it can be helpful to overcome the limitations of bulk hydrate systems, such as poor heat extraction, slow formation, and instability, by also capitalizing on the same fluidity and thermal benefits that made ice and PCM slurries successful in refrigeration and district cooling applications [9].

Gas hydrates are crystalline compounds composed of gas molecules encapsulated within a hydrogen-bonded water framework, that are formed under relatively high-pressure and low-temperature environments. An important group of follow-on developments based on well-established ice water mixes and microencapsulated PCM slurries are CO_2_ hydrate slurries, where solid hydrate particles are suspended in a host fluid (typically water). The resulting two-phase or pseudo-three-phase mixture has improved flowability, enhanced heat-transfer potential, and adaptability to pumped systems. These slurries provide simultaneous utilization of latent heat and sensible heat storage. This made them superior to both conventional PCMs and sensible-only fluids in many thermal management systems [10,11].

CO_2_ forms a structure I hydrate, where each unit cell has up to eight gas molecules trapped in water cages. The dissociation enthalpy of CO_2_ hydrate is in the range of 350 to 520 kJ·Kg^−1^ [12], which is higher than that of ice. But it occurs at temperatures above freezing that is suitable for near-zero or slightly sub-zero thermal applications [13].

CO_2_ clathrate hydrate systems have attracted significant attention as promising candidates for CTES and thermal transport applications due to their relatively high volumetric energy density and favorable phase equilibrium characteristics. However, their practical deployment remains constrained by a combination of fundamental and engineering challenges, including slow formation kinetics, limited heat transfer rates, and difficulties associated with continuous operation under realistic thermofluid conditions. These limitations have driven extensive research into the use of nucleation promoters, surfactants, and thermal conductivity-enhancing additives, as well as the development of advanced reactor configurations and heat exchange systems. From a thermofluids perspective, CO_2_ hydrate slurry systems represent a complex coupling of phase-change thermodynamics, nucleation kinetics, multiphase flow behavior, and heat and mass transfer processes. Hydrate formation is initiated through a nucleation process that can occur either heterogeneously at interfaces or homogeneously within the bulk liquid phase. The nucleation step is highly sensitive to system subcooling, dissolved gas concentration, interfacial area, and hydrodynamic conditions such as mixing intensity and shear rate. Chemical promoters such as tetrahydrofuran (THF), sodium dodecyl sulfate (SDS), and tert-butyl alcohol (TBA) are widely employed to reduce induction time and enhance nucleation reliability by modifying interfacial properties and gas dissolution behavior [14,15].

Despite these improvements, nucleation remains inherently stochastic and difficult to reproduce under dynamic, flow-based conditions, which poses a significant barrier for scalable systems. Following nucleation, hydrate crystal growth and agglomeration govern the evolution of the slurry microstructure. The resulting particle size distribution, morphology, and spatial dispersion directly influence both rheological behavior and thermal transport characteristics. CO_2_ hydrate slurries are typically characterized by non-Newtonian flow behavior, with apparent viscosity strongly dependent on hydrate volume fraction, shear rate, and particle interactions. At low hydrate concentrations (below approximately 5–10 vol%), particles remain well dispersed, and the slurry exhibits near-Newtonian behavior with only modest increases in viscosity. As the hydrate volume fraction increases to intermediate levels (approximately 10–20 vol%), hydrodynamic interactions and particle clustering become significant, leading to shear-dependent viscosity and deviations from Newtonian behavior [16].

At higher hydrate loadings (above approximately 20–30 vol%, the system approaches a densely packed regime where particle-particle interactions dominate, resulting in yield stress behavior, increased flow resistance, and a high risk of agglomeration and plugging [17]. This transition imposes a fundamental limitation on slurry-based systems, as increasing hydrate fractions improve energy storage density but simultaneously degrades flowability and transport efficiency. The thermal transport characteristics of CO_2_ hydrate slurries are similarly governed by the interplay between the dispersed solid phase and the continuous liquid phase. While increasing hydrate content can enhance the effective thermal capacity of the system, its influence on thermal conductivity and convective heat transfer is more complex. In laminar or transitional flow regimes, higher viscosity associated with increased hydrate loading reduces turbulence and can limit convective heat transfer coefficients, offsetting gains in thermal conductivity. Moreover, hydrate formation is an exothermic process, and inadequate heat removal during crystallization can lead to localized temperature rises that reduce the thermodynamic driving force for further hydrate growth. This coupling between heat release, transport resistance, and phase equilibrium represents a key bottleneck in hydrate-based systems [18].

To address these limitations, various strategies have been explored to enhance both kinetics and transport properties. Surfactants and thermodynamic promoters have been shown to reduce induction time and increase gas uptake under specific conditions, although their effectiveness is highly dependent on concentration, reactor configuration, and hydrodynamic regime. Similarly, the incorporation of nanoparticles such as Cu, CuO, Al_2_O_3_, SiO_2_, and carbon nanotubes (CNTs) has been investigated as a means to improve nucleation rates and enhance effective thermal conductivity [15].

Proposed mechanisms include increased surface area for heterogeneous nucleation, improved micro-convection due to Brownian motion, and the formation of conductive percolation networks. However, these effects are not universally beneficial. Experimental studies have shown that enhancements in nucleation kinetics or thermal conductivity do not necessarily translate into improved overall system performance, particularly when increased viscosity or particle aggregation offsets these gains. In addition, issues related to long-term stability, sedimentation, toxicity, and scalability remain largely unresolved, limiting the applicability of such hybrid hydrate–nanofluid systems in practical applications.

Beyond material-level modifications, significant challenges arise at the system level, particularly in the context of continuous hydrate slurry production and transport. Maintaining the thermodynamic conditions required for hydrate formation such as high pressure, low temperature, and controlled gas-liquid mixing under continuous operation is inherently complex. Flow systems must simultaneously ensure sufficient heat removal, uniform mixing, and stable particle suspension, while avoiding sedimentation, agglomeration, and channel blockage. These competing requirements often lead to operational instability, making it difficult to sustain steady-state production and transport of hydrate slurries.

To overcome these challenges, advanced reactor designs such as NETmix reactors have been proposed. Unlike conventional stirred tank reactors that rely on localized mixing from impellers, NETmix systems utilize a network of flow paths to create distributed mixing throughout the reactor volume. This design enhances gas-liquid contact, improves uniformity of hydrate formation, and reduces the likelihood of particle agglomeration and sedimentation. By maintaining more homogeneous flow conditions, NETmix reactors provided improved control over slurry properties and represent a promising approach for continuous hydrate production [19]. Even with improved mixing, achieving a balance between heat removal, particle suspension, and hydrate yield remains a critical challenge.

In parallel, microchannel heat exchangers have emerged as a promising solution for addressing the thermal management limitations of hydrate systems. Due to their extremely high surface-to-volume ratio, microchannels enable rapid heat transfer and efficient removal of the exothermic heat generated during hydrate formation. This allows for better control of local temperature profiles, helping to maintain the thermodynamic driving force for crystallization and prevent the formation of thermal hotspots. Additionally, the confined geometries in microchannels promote higher shear rates and more uniform velocity distributions, which can assist in maintaining particle suspension and reducing sedimentation. These characteristics make microchannel systems particularly attractive for applications requiring compact, high-performance thermal management and continuous operation [20,21].

The use of microchannel systems introduces new challenges associated with slurry handling at small scales. The narrow channel dimensions make the system highly sensitive to particle aggregation and deposition, increasing the risk of clogging and flow blockage. Furthermore, the non-Newtonian rheological behavior of hydrate slurries can lead to unpredictable pressure drops and flow instabilities, particularly under high solid loading conditions. These issues necessitate precise control of slurry properties and operating conditions, as well as the integration of advanced monitoring and control systems to ensure reliable operation [22,23].

Despite the extensive body of literature on hydrate formation kinetics, additives, and reactor design, a critical gap remains in the integrated understanding of CO_2_ hydrate slurries from a thermofluid systems perspective. Existing studies evaluate performance metrics such as induction time reduction, gas uptake, or thermal conductivity enhancement in isolation, without considering the coupled interactions between nucleation behavior, rheology, heat transfer, and flow resistance. In practical systems, these factors are inherently interdependent, and improvements in one aspect may lead to unintended consequences in another. For example, increasing hydrate volume fraction enhances energy storage capacity but significantly increases viscosity and pressure drop, limiting transportability. Similarly, additives that accelerate nucleation may promote particle agglomeration or destabilize the slurry during long-term operation.

This limitation is further compounded by the way prior research has been framed. Much of the literature emphasizes application-level configurations such as microchannel heat exchangers, while fundamental parameters governing hydrate slurry behavior have not been sufficiently highlighted. Among these, hydrate volume fraction emerges as a primary governing variable that simultaneously controls flow characteristics, thermal transport, and storage capacity. Despite its central role, it is rarely treated as a unifying parameter across studies, leading to fragmented conclusions that are difficult to translate into system design.

This work addresses these limitations by presenting a comprehensive, design-oriented review of CO_2_ hydrate slurry systems that explicitly links fundamental phenomena with system-level performance. Unlike prior reviews that focus primarily on kinetics or material enhancements, this study integrates nucleation physics, multiphase flow behavior, and thermal transport with reactor and heat exchanger design considerations. Particular emphasis is placed on hydrate volume fraction as a governing parameter that couples energy storage capacity with flowability and heat transfer performance. In addition, the review critically evaluates the system-dependent effectiveness of additives and highlights the trade-offs associated with their use. By bridging the gap between laboratory-scale observations and engineering implementation, this work aims to provide a framework for the development of scalable, efficient, and reliable CO_2_ hydrate slurry systems for real-world CTES applications.

## 2. CO_2_ Hydrate: Formation and Structure

**Figure 1:**
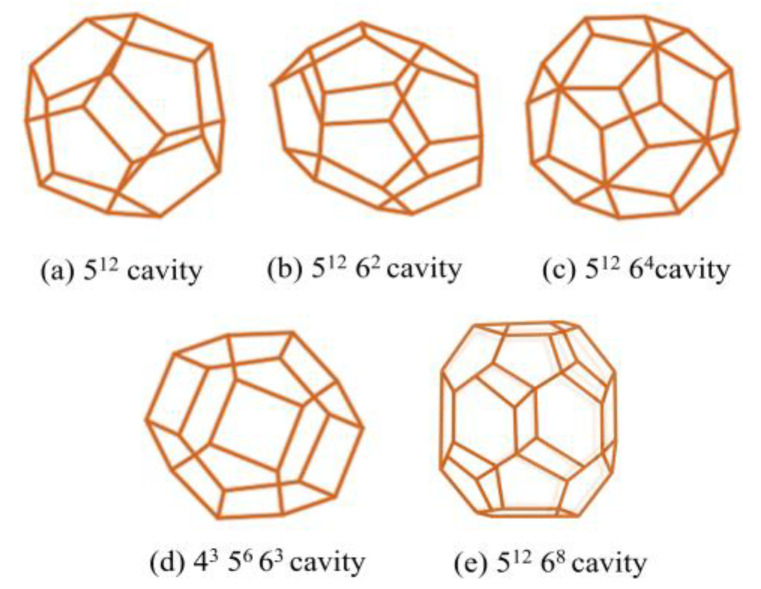
Polyhedral cage structures in gas hydrate formations—(**a**) 5^12^ cavity—sI structure; (**b**) 5^12^6^2^ cavity—sII structure; (**c**) 5^12^6^4^ cavity—sII structure; (**d**) 4^3^5^6^6^3^ cavity—sH structure; (**e**) 5^12^6^8^ cavity—sH structure. Reproduced with permission from published by Elsevier, Amsterdam, The Netherlands, 2006 [25].

Gas hydrates are crystalline clathrates in which hydrogen-bonded water molecules assemble into polyhedral cages that can encapsulate gas molecules. The most common structural frameworks are shown in Figure 1 which are structure I (sI), structure II (sII), and structure H (sH). Structure I consists of 46 water molecules per unit cell, forming two 5^12^ small cages and six 5^12^6^2^ large cages. This framework is typically stabilized by small guest molecules such as CH_4_ or CO_2_. Structure II is larger, containing 136 water molecules per unit cell, with sixteen 5^12^ cages and eight 5^12^6^4^ cages; it accommodates larger guest molecules such as for example propane or iso-butane. Structure H is rarer, requiring a combination of small and large guest molecules, and features a hexagonal lattice with three 5^12^ cages, two 4^3^5^6^6^3^ cages, and one large 5^12^6^8^ cage. Under pure CO_2_-H_2_O conditions, CO_2_ crystallizes exclusively in the sI lattice, with large cages nearly fully occupied and small cages partially filled, leading to hydration numbers between 5.8 and 6.2 depending on temperature and pressure [24].

The thermodynamic framework for hydrate stability is conventionally described by the van der Waals-Platteeuw (vdW-P) model that links cage occupancies to guest fugacity and host lattice chemical potentials. This solid-solution model has been refined to account for multiple occupancy and guest–guest interactions. In the case of CO_2_ hydrate, this model successfully predicts both cage filling and equilibrium conditions when combined with modern equations of state for CO_2_ solubility. The sI framework is particularly favored by CO_2_ due to its molecular size, which fits optimally into the 5^12^6^2^ cages, producing near-unity occupancy in the large cages while allowing only partial filling in the smaller 5^12^ cages [26,27].

Phase equilibrium measurements confirm that CO_2_ hydrates are stable under conditions near 271.6 K and 1.044 MPa, where the four-phase coexistence of vapor, liquid water, ice, and hydrate occurs. These equilibrium points are consistent with laboratory data from stirred reactors. The presence of salt shifts the stability envelope to lower temperatures and higher pressures due to activity suppression of water [28].

The dissociation enthalpy of CO_2_ hydrate is of particular interest when evaluating hydrate slurries for cold thermal energy storage. The latent heat of dissociation of CO_2_ hydrate is in the range of 55–62 kJ·mol^−1^ of guest, corresponding to approximately 350 to 520 kJ·kg^−1^ of water, depending on cage occupancy [1,29].

In comparison, the latent heat of fusion for ice/water is 333 kJ·kg^−1^. This means that per unit mass of water, CO_2_ hydrates can store equal or higher amounts of latent heat than ice, especially when near-complete cage occupancy is achieved. CO_2_ hydrate slurry combines this high enthalpy with favorable transport properties by facilitating ease of pumping as a cold fluid, unlike solid ice slurries, which require mechanical agitation to suspend ice particles [30].

Experimental calorimetric studies shows that CO_2_ hydrate slurries possess an enthalpy of dissociation approximately 20–25% higher than comparable ice slurries under equivalent volumetric fractions [31].

Differential scanning calorimetry (DSC) and T-history method measurements consistently report this advantage of hydrates as more efficient cold thermal energy carriers. The enhancement originates not only from the hydrate dissociation enthalpy but also from the higher gas solubility in water, which sustains hydrate stability at moderately elevated temperatures compared to ice. These properties make CO_2_ hydrate slurries promising candidates for refrigeration, district cooling, and air-conditioning systems where compact and efficient energy storage is required [32].

Nucleation of CO_2_ hydrates remains highly stochastic, with induction times spanning orders of magnitude under seemingly identical conditions. Classical nucleation theory adapted for hydrates describes this behavior by relating the free energy barrier to supersaturation (guest fugacity) and interfacial free energy. Once nucleated, hydrate growth proceeds through transport of CO_2_ across the water-hydrate interface, and this is always enhanced by interfacial stirring. Advanced techniques including Raman spectroscopy, NMR, neutron diffraction, and X-ray diffraction converge on the structural picture of sI CO_2_ hydrate with full occupancy in large cages and variable filling of the smaller cages. In the sI hydrate lattice, each unit cell consists of eight cages typically two small pentagonal dodecahedra (5^12^) and six larger tetrakaidekahedral cages (5^12^6^2^). Because the linear CO_2_ molecule fits more comfortably into the larger cages, these are almost always fully occupied, while the smaller cages are often only partially filled or even left vacant. This uneven distribution arises from differences in the interaction energy between CO_2_ and the water framework, as well as from temperature and pressure conditions that govern molecular stabilization within the lattice. As a result, the formed hydrate departs from the ideal stoichiometric ratio, since the crystalline structure can remain stable even when not all cavities are filled. The extent of cage occupancy directly affects the macroscopic behavior of the solid changes in occupancy modify the unit-cell dimensions, density, and bulk modulus, and are reflected in measurable shifts in the lattice constant and thermal expansion. These microscopic variations in molecular arrangement thus bridge the gap between local structural ordering and the observable thermophysical properties of CO_2_ hydrates [13].

Because guest molecules do not uniformly occupy all hydrate cages under practical formation conditions, deviations from ideal stoichiometry are frequently observed. These occupancy variations yield hydration numbers typically higher than the ideal 5.75, consistent with measured lattice parameters and observed thermal expansion [33].

A distinct feature of CO_2_ hydrate is its metastable preservation behavior on warming. Under near-atmospheric conditions, only a small fraction of gas is released until the system approaches 271 K, at which point dissociation occurs sharply. This behavior is linked to the formation of a protective ice layer and mass-transfer limitations within the hydrate–ice composite shell. Such self-preservation enhances the practical viability of CO_2_ hydrate slurries as cold energy carriers, since thermal losses during short-term storage or transport are minimized compared to ice slurries that undergo continuous melting at temperatures above 273 K [34].

Pure carbon dioxide (CO_2_) usually forms a type of hydrate called structure I (sI). When larger molecules like tetrahydrofuran (THF) or propane are added, they can change the crystal to structure II (sII), which is more stable for these big molecules. In gas mixtures that contain CO_2_ together with methane (CH_4_) or nitrogen (N_2_), the hydrate still keeps the sI form. However, the different gases don’t fill the cages equally and they spread out depending on their size and pressure (fugacity). The bigger cages are usually almost completely filled, while the smaller ones can be partly filled. This uneven filling affects both the stability of the hydrate and how much energy it releases or absorbs when it breaks down. To understand these details better, a Raman spectroscopy is used to study the gas molecules inside the cages and combine the results with van der Waals-Platteeuw (vdW-P) [35] thermodynamic modeling. Together, these tools help explain how mixed-gas hydrates store and release energy, which is important for designing effective hydrate-based thermal storage systems [36].

The dissociation enthalpy of CO_2_ hydrate must be compared with ice only after the normalization basis is clearly defined. In the literature, the enthalpy of CO_2_ hydrate dissociation is commonly reported as 350–520 kJ·Kg^−1^ of guest CO_2_, while the hydration number of sI CO_2_ hydrate varies with cage occupancy and is typically taken between 5.75 for full occupancy and 7.67 when only the large cages are occupied; experimental values around 5.6–6.6 are also widely reported.

Using the relation,

(1) $$\Delta h_{d,water}=\Delta H_{d}/(nM_{H_{2}O})$$

this corresponds to roughly 398–599 kJ kg^−1^ of water incorporated into hydrate, which is higher than the latent heat of fusion of ice (333 kJ·kg^−1^) when both are expressed on a water-phase basis. However, if the same hydrate dissociation enthalpy is normalized by the total hydrate mass rather than the water mass alone, the equivalent value is only about 302–420 kJ kg^−1^ hydrate, showing that the apparent advantage depends strongly on the selected basis [13,29].

For slurry applications, the comparison becomes more restrictive because the relevant metric is not the enthalpy of the pure hydrate phase, but the effective cold storage capacity of the entire slurry, which includes hydrate particles, free water, and dissolved gas. Therefore, a direct statement that CO_2_ hydrate slurry has a universally higher dissociation enthalpy than ice slurry is too broad. A more accurate interpretation is that the hydrate phase can provide a higher phase-change enthalpy than ice on a water-converted basis, while the slurry-level advantage depends on hydrate fraction, phase composition, operating pressure, and the normalization method used in the calorimetric measurement. An overall dissociation enthalpy of approximately 507 kJ·kg^−1^ of initial water has been reported for CO_2_ hydrate–ice mixtures, reflecting the combined contributions of hydrate decomposition and residual ice melting rather than an intrinsic property of the hydrate phase alone. This distinction is critical when interpreting thermal storage performance. Comparisons with ice slurry systems should therefore be presented on a consistent basis, such as per kg of carrier water, per kg of total slurry, or per unit slurry volume, with the hydration number and hydrate volume fraction explicitly specified [37].

## 3. Formation of CO_2_ Hydrate Slurries

### 3.1. Continuous Synthesis of CO_2_ Hydrate Slurry

**Figure 2:**
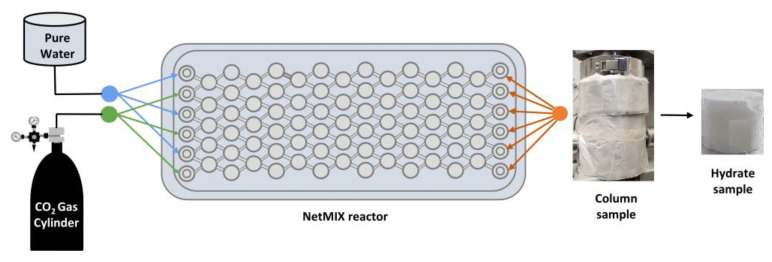
Conceptual schematic representation of the NETmix continuous reactor for CO_2_ hydrate slurry production, illustrating the injection of water and CO_2_ into the mixing column and the formation of hydrate samples. Developed by the authors based on literature descriptions reported in Lozada Garcia et al., *Chemical Engineering Science* **2023** [19].

Continuous production of CO_2_ hydrate slurry is critical for advancing hydrate-based thermal energy storage systems toward real-world implementation. Compared to conventional batch processes, which often suffer from prolonged induction times and inconsistent hydrate quality, continuous systems provide improved process control, scalability, and the potential for near steady-state operation. Continuous hydrate formation can be achieved using advanced reactor configurations that maintain sustained contact between CO_2_ gas and the aqueous phase under controlled temperature and pressure conditions. Among these technologies, microstructure static mixer reactors such as the NETmix device have emerged as promising platforms due to their ability to provide rapid mixing, enhanced heat transfer, and efficient gas-liquid contact [19]. Figure 2 illustrates the NETmix reactor, a continuous static mixer system designed for rapid formation of CO_2_ hydrate slurry. The reactor consists of a network of interconnected chambers and channels that create near-perfect mixing conditions and high interfacial area between the gas and liquid phases. Water and CO_2_ are continuously injected into the reactor through controlled feed streams, where chaotic advection and hydrodynamic instabilities enhance gas-liquid contact and mass transfer. The reactor is equipped with cooling plates that efficiently remove the heat released during hydrate formation, maintaining stable operating conditions. The formed hydrate slurry flows downstream where it can be collected or recirculated depending on the operating mode. This configuration enables fast hydrate formation with short induction times and supports continuous operation due to its high heat and mass transfer performance [19].

Maintaining slurry concentration within an optimal range is essential to prevent excessive solid accumulation, which can lead to rheological instability or flow obstruction. Control strategies that balance hydrate growth rate and slurry extraction rate enable long-duration operation while achieving high conversion efficiency. The continuously produced slurry exhibits uniform particle size distribution and improved flowability, attributes that are highly desirable in downstream applications such as pumped storage loops or heat exchanger systems.

### 3.2. Reactor Configurations for CO_2_ Hydrate Slurry Production

#### 3.2.1. Lab-Scale Synthesis

**Figure 3:**
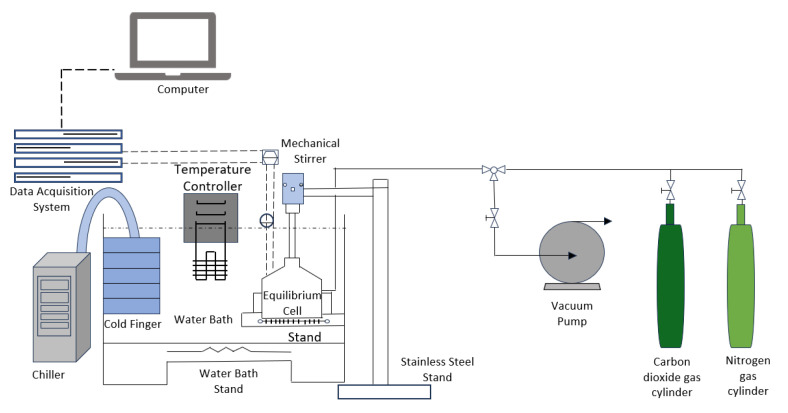
Schematic diagram of experimental setup used for the measurement of CO_2_ hydrate kinetics. Adapted with permission from Ndovu et al., *Ind. Eng. Chem. Res.* **2024**. Copyright © American Chemical Society [38].

At the lab scale, the synthesis of CO_2_ hydrate slurries is primarily conducted using batch reactors or small-scale continuous reactors as shown in Figure 3. These reactors are designed to operate under high-pressure conditions (typically between 3 MPa to 10 MPa) that are necessary for the formation of CO_2_ hydrates. The primary function of these reactors is to facilitate the efficient mixing of CO_2_ gas and water under controlled temperature and pressure conditions. To achieve optimal hydration, the reactors are equipped with high-efficiency agitators that promote the dissolution of CO_2_ into the liquid phase, ensuring the availability of CO_2_ for hydrate formation. The most common configuration in lab-scale setups includes a cylindrical high-pressure reactor with temperature regulation, a stirrer for constant agitation, and a pressure monitoring system to maintain the required conditions [38].

The batch reactor system is relatively simple to set up and is commonly used in initial experimental stages to investigate hydrate formation kinetics, nucleation processes, and the effects of different operating conditions. These reactors typically have a well-defined volume, making them ideal for performing controlled experiments where variables such as CO_2_ concentration, temperature, and pressure are systematically altered to understand their effects on hydrate formation. A typical setup in a batch reactor includes a gas inlet for CO_2_, a liquid inlet for water, a cooling jacket or internal coil heat exchanger to remove heat from the system, and a stirrer to keep the system well-mixed and prevent hydrate aggregation.

For more advanced studies, continuous systems such as Continuous Stirred Tank Reactors (CSTR) are used. These systems facilitate for a continuous flow of CO_2_ gas and water, enabling steady-state hydrate formation. The continuous flow ensures that the system can sustain hydrate formation for extended periods without the need for batch cycles. CSTRs are designed to operate with constant input and output of reactants and products, making them ideal for simulating real-world applications where continuous production of hydrate slurry is required. CSTRs also offer the advantage of uniform slurry characteristics, such as particle size distribution, which is essential for downstream processes like heat extraction. Heat exchangers are also integrated into lab-scale setups to test the thermal performance of the CO_2_ hydrate slurry during its formation and subsequent dissociation. These heat exchangers are often small-scale, allowing researchers to directly measure the thermal conductivity and heat capacity of the hydrate slurry. By integrating heat exchangers into the system, researchers can simulate the thermal response of the slurry, which is crucial for evaluating the efficiency of the hydrate in thermal energy storage applications. In this way, lab-scale reactors serve as versatile platforms for investigating various aspects of CO_2_ hydrate slurry formation, including formation kinetics, thermal behavior, and the effects of different additives or promoters [39].

Despite the advantages of lab-scale systems, they are often limited by the scale-up challenges. The small volume of these reactors can lead to fluctuations in CO_2_ flow rates and heat transfer, which may not reflect the performance of large-scale systems. Achieving consistent slurry quality and preventing the aggregation of hydrate particles can be difficult, especially when scaling up to industrial applications. As a result, while lab-scale synthesis provides valuable insights into hydrate formation, it is not always directly translatable to industrial-scale systems.

#### 3.2.2. Industrial Scale Fabrication

**Figure 4:**
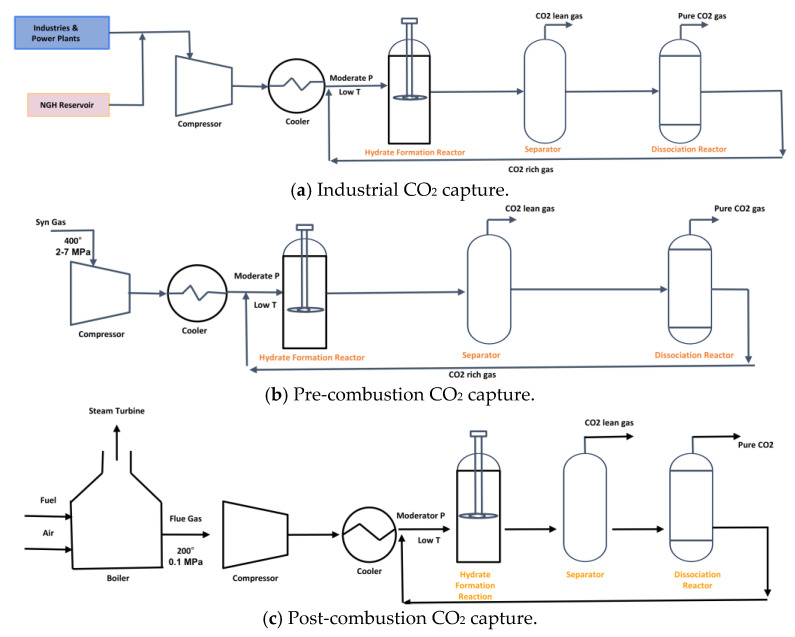
Conceptual process schematics of semi-clathrate hydrate-based CO_2_ capture pathways illustrating (**a**) industrial gas streams, (**b**) pre-combustion capture, and (**c**) post-combustion capture, including compression, cooling, hydrate formation, separation, and dissociation stages. Developed by the authors based on literature reported by Rukh et al., *Carbon Capture Science & Technology* **2024**, under the Creative Commons Attribution-Noncommercial-No Derivatives 4.0 International License (CC BY-NC-ND 4.0) [40].

At the industrial scale, the production of CO_2_ hydrate slurries is far more complex and requires robust equipment designed to handle continuous production under high-pressure conditions. These systems are designed to meet the demands of large-scale applications such as cold storage, cooling systems for industrial processes, and energy storage. The equipment used for industrial-scale slurry production must be capable of operating for extended periods without significant wear and tear, as well as handling larger volumes of CO_2_ and water. One of the primary equipment types used in industrial-scale systems is the Continuous Stirred Tank Reactor (CSTR). Figure 4 shows CSTRs used in industrial-scale applications are much larger and more sophisticated than their lab-scale counterparts. They are designed to accommodate a higher throughput of CO_2_ and water, ensuring that the reaction occurs at a constant rate, and that slurry production is stable over time [40].

Process configurations for semi-clathrate hydrate-based CO_2_ capture applied to industrial streams, pre-combustion gas, and post-combustion flue gas as shown in Figure 4. In all three cases, the CO_2_-containing gas stream is first conditioned through compression and cooling to reach moderate pressure and low temperature conditions required for semi-clathrate hydrate formation. The conditioned gas is then introduced into a hydrate formation reactor containing an aqueous solution of quaternary ammonium or phosphonium salt promoter. Under these conditions, CO_2_ is preferentially incorporated into the semi-clathrate hydrate lattice due to favorable thermodynamic stability relative to other gas components.

The outlet stream from the hydrate reactor enters a separator where the unreacted CO_2_-lean gas phase is withdrawn. The CO_2_-rich hydrate slurry is transferred to a dissociation reactor, where controlled heating or pressure reduction destabilizes the hydrate structure and releases high-purity CO_2_. The regenerated promoter solution is recycled back to the hydrate formation reactor, and this enables continuous operation.

In the industrial configuration, CO_2_ from process gas streams is treated directly. In the pre-combustion scheme, syngas at elevated temperature and pressure is first cooled and then subjected to hydrate formation for selective CO_2_ removal. In the post-combustion route, flue gas from the boiler is compressed and cooled before hydrate formation. These configurations demonstrate the flexibility of semi-clathrate hydrate technology for integration into different carbon capture pathways.

The industrial-scale CSTR is often coupled with high-efficiency heat exchangers to maintain the required temperature conditions for hydrate formation. The heat exchangers are designed to remove the heat generated during the exothermic formation of CO_2_ hydrates, preventing the temperature from rising beyond the optimal range for hydrate growth. Cooling systems are critical in industrial-scale systems, as they help regulate the temperature within the reactor to ensure that the CO_2_ hydrate crystals grow efficiently. The design of the heat exchangers and cooling systems must take into account the large scale of the system and the need for constant heat removal to maintain steady operation [41].

In addition to CSTRs, industrial systems often incorporate specialized gas-liquid separation units. These units separate unreacted CO_2_ gas from the hydrate slurry, allowing for the recycling of CO_2_ back into the reactor. This separation process is essential for maintaining system efficiency and reducing the overall cost of operation. In some systems, CO_2_ gas is collected and reintroduced into the reactor to maintain the required concentration of CO_2_ in the aqueous phase. This closed-loop system helps minimize CO_2_ waste and maximizes the amount of hydrate produced over time. Another important aspect of industrial-scale systems is slurry management. As CO_2_ hydrate particles are formed, they must be continually circulated and pumped through the system to prevent settling and aggregation. Industrial systems are equipped with slurry pumps, which help maintain consistent flow and prevent the formation of large hydrate clusters that could clog pipes or heat exchangers. To address issues of sedimentation and particle aggregation, industrial-scale systems may incorporate additional components such as slurry stabilizers or dispersants. These chemicals help prevent the formation of large hydrate particles, ensuring that the slurry remains flowable and easy to pump through the system [42].

The industrial-scale fabrication of CO_2_ hydrate slurry systems also involves extensive safety measures, as the high pressures and temperatures involved in the reaction can be hazardous. Industrial systems need to be equipped with safety valves, pressure relief systems, and redundant safety measures to prevent over-pressurization or equipment failure. The use of corrosion-resistant materials is critical in ensuring the longevity and reliability of the system, as the high-pressure conditions and aggressive chemical environment can lead to rapid wear and degradation of equipment components. As such, industrial-scale fabrication requires careful consideration of materials, safety systems, and operational protocols to ensure smooth and reliable operation [43].

In practice, hydrate-based cold thermal energy storage and slurry cooling technologies are being explored across a wide range of industrial and mission-critical applications. Large-scale district cooling and seasonal cold storage systems can utilize hydrate storage tanks to shift cooling loads and improve energy efficiency during peak demand periods. In the food processing and refrigerated warehousing industries, hydrate slurry systems provide rapid cooling capability and improved temperature uniformity compared to conventional secondary refrigerants. Hydrate-based cooling is also being investigated for high-performance data centers, where maintaining stable thermal conditions and handling high heat fluxes are essential for reliable operation. In defense and aerospace applications, compact hydrate slurry cooling systems offer potential advantages for thermal management of electronics, mobile cooling units, and field-deployable refrigeration systems due to their high energy density and operational flexibility. But for systems that need compact equipment, fast response, and compatibility with fluid-based heat exchangers, slurry systems offer far better performance. Recognizing these contrasts in heat-transfer rate, storage density, operating stability, and system cost is key to selecting the right approach for a given thermal-energy-storage or refrigeration application.

### 3.3. Bulk Hydrate Systems

Bulk hydrate systems are considered for large-scale thermal storage due to their high latent heat storage capacity. These systems involve the formation of hydrate masses in the form of solid cakes or beds. The advantage of bulk systems lies in their ability to store large amounts of energy in a small volume and this makes them ideal for applications requiring high energy density. This storage density comes at a cost: bulk hydrate systems suffer from significant thermal resistance due to the low thermal conductivity of the hydrate solids. As hydrate crystals form, they create an insulating layer around the hydrate mass, which impedes the efficient transfer of heat. This thermal resistance can reduce the overall efficiency of a system, leading to slower heat extraction and reduced performance in real-world applications.

Bulk hydrate systems require mechanical methods to continuously extract the stored cold energy. These methods typically involve either melting the hydrate bed or mechanically removing the hydrate to release the stored thermal energy. Both of these processes add complexity and cost to the system, as they require additional equipment and energy input. Mechanical removal of hydrates can also result in physical degradation of the hydrate crystals, reducing their efficiency for subsequent cycles. Another challenge with bulk hydrate systems is their sensitivity to changes in temperature and pressure. As the hydrate mass forms and dissociates, it is essential to maintain stable conditions to prevent the formation of insulating layers or the degradation of the hydrate structure. In large-scale systems, these fluctuations can be difficult to control, especially when the system is subject to external temperature changes or fluctuating energy demands. The need for large tanks or vessels to accommodate the hydrate mass means that bulk hydrate systems can be cumbersome and difficult to integrate into smaller or mobile applications [44].

### 3.4. CO_2_ Hydrate Slurry Systems

Hydrate slurry systems have several advantages over bulk hydrate systems, primarily due to their ability to circulate and pump the hydrate particles within a continuous fluid phase. The slurry’s high surface area-to-volume ratio allows for much more efficient heat transfer, making hydrate slurries ideal for applications requiring fast thermal response, such as refrigeration and on-demand cooling systems. The fine particles of hydrate within the slurry allow for a large surface-area contact with the surrounding fluid, enabling rapid heat absorption and dissipation [45].

One of the key benefits of hydrate slurries is their flexibility in terms of system design. Unlike bulk hydrate systems that need large vessels or beds to store the hydrate mass, slurry systems can be integrated into compact heat exchangers and microchannel geometries. Hydrate slurries can be continuously pumped and circulated through heat exchangers, allowing for dynamic heat extraction and cooling. The continuous operation of slurry systems enables more efficient use of resources, as the slurry can be continuously replenished with CO_2_ gas without the need for extensive reformation cycles.

### 3.5. Comparison of CO_2_ Hydrate Slurry Versus Bulk Hydrate Systems

The choice between a slurry-based hydrate system and a bulk hydrate system mainly depends on two things: how quickly heat can be moved in and out of the hydrate, and how much thermal energy can be stored in a given volume. In a bulk system, the hydrate usually forms as a solid block or bed, converting a large portion of water into hydrate. This allows a high amount of latent heat to be stored within a small space and is a clear advantage when energy density matters. The drawback, however, appears as soon as the hydrate layer thickens. Once the solid structure develops, its thermal conductivity drops sharply. The hydrate crystals, together with small gas or water pockets trapped within, act like insulation layers. This makes it harder for heat to flow during both formation and melting. As the process continues, thermal resistance grows, the active surface area for heat exchange decreases, and the system begins to respond sluggishly [39].

In contrast, a slurry hydrate system behaves more like a moving suspension, with fine hydrate particles dispersed in a liquid carrier. Because these particles stay mobile and can circulate through the system, the contact between the solid hydrate and the surrounding fluid is constantly renewed. The large surface area that results enables heat to be absorbed and released much faster. This is why slurry systems are better suited for processes that require quick cooling or frequent thermal cycling, such as refrigeration and on-demand cold storage. The trade-off is that slurry systems store slightly less energy per unit volume, since part of the volume is occupied by the carrier liquid and any unreacted gas. They can also face practical issues such as sedimentation, clogging, or particle agglomeration when flow conditions are not well controlled [46].

### 3.6. Critical Assessment of Experimental Approaches and Current Challenges in CO_2_ Hydrate Slurry Systems

A critical evaluation of the current literature shows that the reported performance of CO_2_ hydrate slurry systems is strongly dependent on the experimental approach, and results obtained under different configurations are not directly comparable. In stirred batch reactors, hydrate formation kinetics are highly influenced by hydrodynamic conditions such as mixing intensity, impeller design, and gas-liquid dispersion. Variations in stirring conditions and reactor configuration can significantly alter apparent formation rates, indicating that hydrate kinetics in such systems are strongly coupled with heat and mass transfer effects rather than representing purely intrinsic nucleation behavior [47]. Consequently, induction time and gas uptake data reported across different batch studies may reflect differences in reactor hydrodynamics rather than differences in material or additive performance.

In contrast, dynamic-loop systems provide a more realistic representation of hydrate slurry transport and reveal additional limitations associated with rheology and particle interactions. Hydrate slurries exhibit non-Newtonian behavior, with viscosity increasing significantly as hydrate volume fraction increases and shear rate decreases [16]. These systems also highlight the strong tendency of hydrate particles to agglomerate, leading to increased pressure drop and potential flow blockage under low-shear conditions. The presence of additives can influence not only hydrate formation kinetics but also interparticle interactions and flow behavior. In particular, certain additives can reduce agglomeration and improve slurry mobility, demonstrating that evaluation based solely on induction time is insufficient for assessing overall system performance [48].

Continuous reactor configurations offer an alternative approach by improving gas–liquid contact and enhancing heat removal during hydrate formation. Improved mixing and thermal management in such systems can lead to more stable and efficient hydrate production [19]. However, while continuous operation can enhance formation rates, it introduces additional challenges related to slurry handling, particle deposition, and long-term operational stability. This indicates that improvements in formation kinetics alone do not guarantee practical applicability unless issues related to slurry transport and stability are also addressed.

The role of additives in hydrate systems must therefore be interpreted within this broader context. Nanoparticle-based approaches do not consistently lead to improved performance. The influence of nanoparticles on hydrate formation depends strongly on particle concentration, interaction with surfactants, and dispersion characteristics, and does not always result in proportional increases in gas consumption or storage capacity [49]. Similarly, graphene oxide has been shown to reduce induction time significantly; however, its effectiveness is highly dependent on concentration and formulation, with optimal performance occurring within a limited operating range [50]. These findings indicate that nanoparticle enhancement is highly system-specific and cannot be generalized across different experimental conditions.

Surfactant-based promoters also exhibit complex behavior that extends beyond simple kinetic enhancement. In addition to influencing formation kinetics, surfactants can modify hydrate morphology, which in turn affects particle aggregation and slurry flow characteristics [51]. Comparative studies further demonstrate that alternative promoters can outperform conventional surfactants under certain conditions, indicating that promoter effectiveness depends on system-specific factors rather than a single universal mechanism [52].

Surfactant- and nanoparticle-assisted promotion of CO_2_ hydrate formation should not be interpreted as universally beneficial, because the reported improvement is strongly dependent on additive concentration, pressure, hydrodynamics, and the performance metric used. Studies on surfactant systems show that sodium dodecyl sulfate (SDS) can provide strong kinetic enhancement, with clear reduction in induction time and faster hydrate growth relative to pure water systems, indicating that interfacial effects can accelerate nucleation when gas–liquid contact is the controlling limitation. However, this behavior is not monotonic across all conditions. Investigations involving SDS and silver nanoparticles show that neither additive alone consistently decreases induction time or significantly increases storage capacity under all operating conditions, while combined SDS-nanoparticle systems primarily improve overall gas uptake rather than uniformly accelerating the onset of nucleation. This indicates that a promoter may enhance hydrate growth and storage capacity without proportionally improving nucleation kinetics, and that results obtained under one reactor configuration cannot be directly generalized to others [53].

Surfactants and nanoparticles are widely reported as kinetic promoters for CO_2_ hydrate formation, yet their effectiveness depends strongly on formulation and operating conditions. Graphene oxide (GO) systems demonstrate that performance improvements occur only within a limited concentration range. At 279 K and 3–5 MPa, GO reduces induction time by approximately 53–74% and increases gas consumption by about 5–16%, with optimal performance observed at low concentrations. The enhancement does not increase monotonically with concentration, indicating that higher particle loading does not necessarily lead to improved hydrate formation and may instead introduce dispersion or transport limitations [54].

From a transport perspective, kinetic improvement alone is insufficient to evaluate additive performance in hydrate slurry systems. Flow-loop studies show that CO_2_ hydrate slurry formed in pure water exhibits shear-thickening behavior and a strong tendency toward blockage, whereas systems containing non-ionic surfactants such as Tween-80 exhibit shear-thinning behavior and remain flowable under comparable conditions. This difference is linked to hydrate morphology, where surfactant-assisted systems promote hydrate formation around dispersed gas bubbles and reduce particle adhesion to solid surfaces. Additional studies demonstrate that low concentrations of SDS improve slurry homogeneity and reduce rapid agglomeration, although rheological behavior remains sensitive to operating conditions. High-pressure rheological measurements further confirm that SDS exhibits an anti-agglomeration effect over a range of pressures and significantly alters slurry behavior during both hydrate formation and dissociation [55].

These observations indicate that the most relevant criterion for additive selection in CO_2_ hydrate slurry systems is not limited to induction time reduction, but must include the combined effects of hydrate fraction, apparent viscosity, agglomeration tendency, and resistance to blockage. Differences between studies therefore reflect variations in experimental constraints rather than contradictions in the underlying mechanisms. Surfactants primarily influence interfacial tension, gas dispersion, and particle adhesion, whereas nanoparticles contribute mainly through heterogeneous nucleation sites and localized heat transfer pathways. Depending on whether the system is quiescent, mechanically stirred, or operated under continuous flow conditions, the same additive may exhibit significantly different performance. For this reason, additive effectiveness in CO_2_ hydrate systems should be evaluated using at least three criteria: nucleation behavior, total gas uptake, and slurry rheology. A promoter that improves only one of these aspects may not be suitable for practical cold thermal energy storage or hydrate slurry transport applications.

In practical hydrate-based cooling or thermal energy storage systems, reactor and heat exchanger design must address the strong heat and mass transfer limitations associated with hydrate crystallization. The formation of CO_2_ hydrate is an exothermic process with a heat of formation typically reported in the range of 350 to 520 kJ. Kg^−1^ of CO_2_ incorporated into the hydrate lattice [56].

If this heat is not removed efficiently, the local temperature increase can suppress further hydrate nucleation and growth. In conventional stirred batch reactors, hydrate crystals often form at the gas-liquid interface and create a hydrate film that acts as a diffusion barrier, reducing the rate of gas transport into the liquid phase [57].

**Figure 5:**
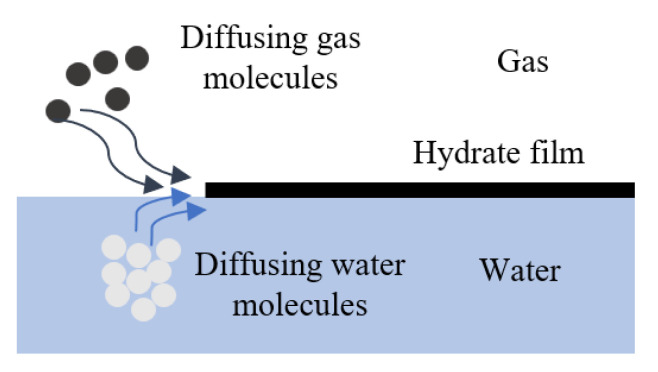
Schematic representation of hydrate film formation at the gas-liquid interface, illustrating diffusion of gas and water molecules and the development of a hydrate layer that acts as a mass transfer barrier. Redrawn and adapted from Kar et al. [58].

While these criteria are essential for evaluating additive performance, they do not address the fundamental transport limitations that govern hydrate formation at the system level. Figure 5 illustrates that hydrate formation at the gas–liquid interface leads to the development of a solid hydrate film that separates the gas and liquid phases. Once this film is made, further hydrate growth becomes controlled by the diffusion of gas molecules through the hydrate layer and the transport of water molecules toward the reaction interface. This additional mass transfer resistance significantly reduces the rate of hydrate formation and can lead to incomplete conversion under poorly mixed or thermally constrained conditions.

For this reason, intensified heat exchanger configurations such as microchannel heat exchangers have been proposed for hydrate-based cooling systems. Microchannel devices typically have hydraulic diameters between 100 and 1000 μm, which produce extremely high surface-area-to-volume ratios and enable heat transfer coefficients exceeding 1–5 kW m^−2^ K^−1^, significantly higher than those obtained in bulk reactor systems. The enhanced thermal control provided by these compact geometries helps maintain stable subcooling conditions during hydrate formation and prevents localized overheating that can inhibit crystal growth [59,60].

Continuous hydrate reactors and slurry circulation loops have also been investigated to overcome agglomeration and transport limitations that occur in static reactors. Experimental flow-loop studies have shown that hydrate slurries can remain pumpable when the hydrate volume fraction is typically maintained below approximately 20–30%**,** while higher hydrate concentrations can lead to rapid increases in apparent viscosity and particle agglomeration that may result in flow blockage [61].

The presence of surfactants such as sodium dodecyl sulfate (SDS) has been shown to improve slurry homogeneity and reduce particle adhesion within circulation loops, allowing hydrate particles to remain dispersed during transport through pipelines and heat exchangers. Engineering reactor concepts therefore often combine enhanced gas dispersion, controlled shear conditions, and compact heat transfer surfaces to sustain hydrate formation under continuous operation. Designs incorporating static mixers, spiral channels, or micro structured heat exchanger plates have been explored to increase gas-liquid interfacial area while simultaneously removing reaction heat and preventing hydrate film formation at interfaces. These engineering considerations demonstrate that the feasibility of hydrate-based cooling systems depends strongly on the integration of reactor geometry, flow regime, and heat exchanger design, rather than relying solely on chemical additives to accelerate hydrate formation [62].

### 3.7. Recovery and Recycling of Materials in Continuous Slurry Production

Continuous CO_2_-hydrate slurry operation requires material management strategies for kinetic/thermal promoters (e.g., surfactants) and dispersed solids (e.g., nanoparticles) so that make-up rates and effluent loads remain low. In hydrate systems, a variety of substances such as for example sodium dodecyl sulfate (SDS) are used. They are typically effective in the 100–500 ppm range, with several studies also reporting activity around 1000 ppm depending on gas, driving force, and matrix; designing loop to retain and recirculate this concentration band minimizes fresh-chemical demand [63].

To improve thermal conductivity of slurry, highly conductive additives and nano powders are used. For these slurries, the following two ways of separation are practical at the process scale. At first, pressure-driven microfiltration and ultrafiltration membranes placed downstream of the gas-liquid separator can recover colloids without breaking the hydrate loop. Ceramic and polymer UF with cutoffs 10–100 nm routinely retain SiO_2_/TiO_2_ and metal colloids; open studies report UF pores 40 nm capturing SiO_2_ dispersions and MF at 180 nm for coarser fractions, with nanomaterial-modified membranes showing higher flux recovery and lower fouling important for 24/7 operation. The second approach is a hydrocyclone. This is a simple device that uses spinning motion to separate heavier solid particles from lighter liquid or slurry without any moving parts. The flow enters tangentially by creating a vortex and heavy particles move to the wall and exit through the bottom (spigot), while lighter fluid or fine slurry exit through the top (vortex finder). In a continuous hydrate process, a hydrocyclone can do more than just remove unwanted solids. It also helps recover and recycle the hydrate-rich portion of the slurry back into the loop. This keeps most of the useful hydrate in circulation while letting out mineral fines or agglomerates that would otherwise clog the system. By doing so, the process stays stable, pressure losses are minimized, and hydrate material isn’t wasted between cycles. In short, the hydrocyclone acts as both a solids separator and a recovery unit, improving the reliability and efficiency of continuous CO_2_-hydrate operation [64].

## 4. Conditions Facilitating the Formation Process, Its Mechanisms, and Nucleation-Enhancing Additives

The formation of CO_2_ hydrate slurries involves the crystallization of gas hydrate particles within a liquid phase and their subsequent suspension in a flowing or stirred medium. This process bridges the disciplines of phase-change thermodynamics, multiphase flow, and applied heat transfer. From a thermofluidic standpoint, understanding the conditions that govern hydrate formation and the transition from bulk hydrate to slurry is essential for optimizing system performance in cold thermal energy storage (CTES) and refrigeration applications.

As discussed earlier, CO_2_ hydrates form when gaseous or liquid carbon dioxide comes into contact with water under suitable thermodynamic conditions typically at pressures above 2.5 MPa and temperatures below 2 °C and 10 °C [65]. The exact phase equilibrium depends on the purity of water and the presence of kinetic promoters. Hydrate formation generally follows a three-step mechanism: gas dissolution into the water phase, nucleation of hydrate crystals. The initial stage involves the local structuring of water molecules around gas molecules to the formation of short-lived hydrate-like clusters. These clusters are highly dynamic and may either dissolve or stabilize depending on the local environment and the presence of stabilizing agents. Once a critical cluster size is reached, it can evolve into a stable hydrate nucleus and initiate crystal growth. One of the key experimental observations associated with this process is the induction time, defined as the time interval between the system entering hydrate-forming conditions and the actual detection of hydrate crystals. This delay can be influenced by multiple factors including temperature, pressure, gas concentration, and the physical characteristics of the reactor system. The nucleation process is also strongly influenced by the presence of additives or promoters. The nucleation of gas hydrates is a stochastic process that marks the onset of hydrate phase formation from a metastable gas-liquid solution. It is typically classified as either homogeneous or heterogeneous nucleation [65].

Homogeneous nucleation refers to the spontaneous formation of hydrate crystals in a uniform and impurity-free aqueous phase without the influence of external surfaces or particles. This process occurs when water molecules and gas molecules randomly align in a way that leads to the formation of hydrate-like structures. Because no surfaces are present to reduce the energy threshold, the initiation of homogeneous nucleation requires significantly higher levels of supersaturation and undercooling compared to heterogeneous nucleation. Homogeneous nucleation is primarily a theoretical or computational concept in the study of hydrate formation. In practical experiments, achieving the stringent conditions necessary for this form of nucleation is extremely challenging, making it rare under standard laboratory or field conditions. The nucleation typically involves a very long induction period, as the likelihood of spontaneous and sufficient molecular alignment in the bulk phase is low. These limitations are why hydrate formation in most engineered systems is dominated by heterogeneous nucleation pathways [66].

Heterogeneous nucleation occurs when gas hydrate crystals begin to form on foreign surfaces such as solid walls, particles, or interfaces within the fluid. This process is thermodynamically favored over homogeneous nucleation because it reduces the critical energy barrier required for the formation of stable nuclei. In practice, most hydrate formation in laboratory and industrial systems initiate heterogeneously. Surfaces such as the walls of the reactor or pre-existing particles in the liquid act as nucleation catalysts by providing sites where the gas and water molecules can be more easily organized into hydrate structures. The presence of a solid substrate lowers the interfacial energy and creates an environment where clusters of gas and water molecules can become stabilized, thereby promoting the formation of critical-sized nuclei. Factors such as surface roughness, chemical composition, wettability, and contact angle directly influence the efficiency of these nucleation sites. For example, hydrophilic surfaces have been observed to promote better contact with water molecules, enhancing the probability of hydrate nucleation. The presence of surfactants or nanoparticles further alters the interfacial characteristics, often enhancing the number and quality of nucleation sites available. In engineered systems, optimizing these surface properties is considered a viable strategy to control and improve hydrate formation kinetics [67].

## 5. Enhancement of Nucleation Processes

The incorporation of chemical additives into the hydrate formation process is a widely used strategy to enhance formation kinetics, control morphology, and enable operation under milder pressure and temperature conditions. These additives are broadly classified into two categories: thermodynamic promoters and kinetic promoters. The formation of gas hydrates typically requires low temperatures and high pressures to stabilize the clathrate structure. Thermodynamic promoters are substances that facilitate hydrate formation by shifting the equilibrium conditions allowing hydrate formation at lower pressures and/or higher temperatures. This modification is achieved by stabilizing the hydrate structure through the incorporation of promoter molecules. These molecules reduce the free energy barrier for hydrate formation. Consequently, these additives are of significant interest in hydrate-based applications, where operational feasibility depends on milder formation conditions. Kinetic promoters improve hydrate formation by reducing induction time and accelerating nucleation without changing equilibrium conditions. Surfactants such as SDS enhance gas-liquid contact and stabilize the interface and support faster and more reliable hydrate growth.

### 5.1. Thermodynamic Nucleation Promoters

A prominent thermodynamic promoter is tetrahydrofuran (THF), which serves as a self-forming agent for structure II (sII) hydrates. In this context, a self-forming agent refers to a large guest molecule capable of occupying the large hydrate cages independently to stabilize the structure. While pure CO_2_ forms sI hydrates, in binary THF + CO_2_ systems, the hydrate transitions to sII, where THF molecules occupy the large 5^12^6^4^ cages and CO_2_ molecules reside in the small 5^12^ cages. This co-occupancy behavior allows hydrate formation at significantly milder conditions than those needed for pure CO_2_ hydrates. The shift in the phase equilibrium boundary toward higher temperatures and lower pressures relative to pure CO_2_ systems indicates enhanced thermodynamic stability [68,69].

Thermodynamic promoters significantly influence the phase equilibrium behavior of CO_2_ hydrates by altering the pressure-temperature (P-T) conditions under which hydrate formation occurs. Typically, CO_2_ hydrates form at high pressures and low temperatures, limiting their practical use in industrial systems. But, the inclusion of promoters such as tetrahydrofuran (THF), cyclopentane (CP), or tetrabutylammonium bromide (TBAB) shifts the phase boundary to higher temperatures and lower pressures. This means that, for a constant temperature, hydrate formation occurs at a lower pressure, or for a continuous pressure, at a higher temperature, thereby expanding the feasible operational window. For instance, in binary hydrate systems like THF + CO_2_, the promoter occupies the large cages of the sII hydrate structure, while CO_2_ fills the small ones. The effect of thermodynamic promoters on hydrate equilibrium conditions is summarized in Table 1.

### 5.2. Kinetic Nucleation Promoters

While thermodynamic promoters shift the hydrate phase equilibrium to more favorable conditions, kinetic promoters enhance the rate of hydrate formation without altering the underlying equilibrium boundaries. They achieve this by promoting faster nucleation, reducing induction time, increasing gas-liquid contact efficiency, and stabilizing the hydrate water interface. These additives are particularly valuable in systems where rapid hydrate formation is critical, such as in cold thermal energy storage, gas separation, and transportation applications.

A widely studied class of kinetic promoters includes surfactants, such as sodium dodecyl sulfate (SDS), cetyltrimethylammonium bromide (CTAB), and nonionic agents like Tween 80. These surfactants reduce the surface tension at the gas-liquid interface, allowing gas molecules to more readily dissolve and diffuse into the aqueous phase where hydrates can form. The presence of surfactants also prevents the agglomeration of hydrate particles by dispersing them uniformly in the fluid. This helps to maintain slurry flowability and increases overall gas uptake. Among these, SDS is particularly effective for CO_2_ hydrate formation. At low concentrations (e.g., 0.05–0.1 wt%), SDS has been shown to significantly reduce the induction time and increase the total amount of gas enclathrated [69].

Further enhancement of hydrate formation can be achieved by using gas mixtures containing CO_2_ as a main component. Mixed-gas systems reshape the early steps of hydrate formation in a way that single-gas systems cannot achieve. The presence of two guests changes the way water molecules align before the first cavity forms. CO_2_ alone demands a narrow set of structural conditions, which makes the early stage fragile. A second gas relaxes these demands by allowing the water network to settle into a wider range of patterns that still lead to hydrate growth. This broadens the range of stable structural configurations available during nucleation [77].

The formation of gas hydrates is controlled by what happens during the earliest moments of nucleation. Before any visible hydrate appears, the system enters a waiting stage where water molecules attempt to organize around gas molecules. This stage is called the induction period. A long induction period means the system is struggling to create the first stable hydrate nucleus. Even though the pressure and temperature fall inside the hydrate region, the water and gas do not immediately form solid hydrate. Instead, small hydrate-like clusters appear briefly and then fall apart. They fail because they have not reached the critical size needed to grow on their own. In the case of CO_2_ hydrates, this delay can be very long and unpredictable. CO_2_ is a linear molecule with a strong quadrupole moment. It requires a very specific water arrangement before a cage can close around it. Most early clusters never survive long enough to become stable. Molecular-level simulations show this clearly. The energy barrier for CO_2_ hydrate nucleation can reach values above 60–100 kT, which makes these early attempts highly unstable. This explains why CO_2_ often sits in a metastable state for a long time before the first solid hydrate appears [66].

When for example, methane is added to the system, the entire nucleation behavior changes. Methane is compact and spherical, so water molecules do not have to align with the same precision required for CO_2_. The water network can form cage-like structures more easily and with more flexibility. Because of this, many more clusters survive past the early stages. The system no longer relies on a single narrow molecular pathway. Several parallel routes appear, each providing a lower barrier toward hydrate formation. As a result, the induction time becomes shorter and more consistent. CO_2_-CH_4_ mixtures typically begin forming hydrates at an earlier stage and with far less delay than pure CO_2_ systems. This improvement has been observed in both simulation and experimental studies, where hydrate growth begins promptly once the mixture reaches the stability zone.

Propane has an even stronger influence, especially when combined with methane. Propane naturally stabilizes the large cavities of structure-II hydrates. When these large cavities appear early in the process, the water network becomes more organized and more capable of supporting further cage formation. Once propane occupies the large cavities, methane quickly enters the small ones. This cooperative behavior removes many of the early obstacles that usually slow down nucleation. In CH_4_-C_3_H_8_ systems, hydrate formation often begins immediately after the system crosses the equilibrium line. The induction time becomes almost negligible because the system does not need to wait for the slow, sequential steps that govern single-gas nucleation. Experiments in porous media show this effect clearly: when methane and propane are present together, hydrate formation begins without the long hesitation typically seen in pure-gas systems [78]. Additional large guest molecules such as cyclopentane have been shown to modify the nucleation pathway of CO_2_ hydrates by stabilizing structure-II hydrate cages during the earliest clustering stages. In mixed CP-CO_2_ systems, cyclopentane preferentially occupies the large 5^12^6^4^ cages and facilitates nucleus stabilization while promoting the persistence of early hydrate clusters. Experimental kinetic measurements show that the presence of cyclopentane reduces induction time and promotes earlier onset of hydrate growth by facilitating interfacial nucleation and improving structural organization of the surrounding water network. Recent studies also show that systems combining cyclopentane with solid nucleation centers can reduce induction time and confirm the strong role of large-guest molecules in accelerating hydrate nucleation kinetics [79,80,81].

### 5.3. Amino Acids—Biobased Kinetic Promoters

Amino acids have recently emerged as effective and eco-friendly kinetic promoters for gas hydrate formation, including CO_2_ hydrates. These naturally occurring, biodegradable molecules possess both hydrophilic and hydrophobic functional groups, enabling them to interact with water and gas phases and facilitate the nucleation process without significantly altering the hydrate’s thermodynamic conditions. Unlike conventional surfactants, which often pose environmental risks, amino acids offer a sustainable alternative while exhibiting significant promotional effects in hydrate systems. In particular, the study evaluated the performance of several amino acids, L-methionine, L-phenylalanine, L-leucine, and glycine, under pressurized CO_2_ environments. The experimental results demonstrated that amino acids such as L-methionine and L-phenylalanine notably enhanced the formation kinetics of CO_2_ hydrates. These compounds reduced induction times and increased overall gas uptake compared to the control system without additives. For example, L-methionine shortened the induction time to nearly one-third of that in the pure water system. This improvement is attributed to the amphiphilic molecular structure of amino acids, which enables them to localize at the gas-liquid interface. Their hydrophobic side chains, such as the methylthioether group in methionine or the aromatic ring in phenylalanine, may provide preferential sites for initial water structuring and CO_2_ diffusion, thereby promoting nucleation [82].

In practice, hydrate nucleation rarely occurs homogeneously. Instead, it typically initiates on reactor walls, solid particles, or gas-liquid interfaces highlighting the importance of heterogeneous nucleation. The induction time is the delay between reaching favorable conditions and the onset of hydrate formation and this varies significantly based on subcooling, agitation, interfacial area, and promoter presence [65]. Surfactants such as sodium dodecyl sulfate (SDS) are commonly used to reduce surface tension and promote rapid hydrate formation. SDS concentrations in the range of 5 to 500 ppm can also reduce induction time by 4.5 h to 8 min [65,83].

Hydrate formation can be achieved using semi-batch stirred tank reactors, loop flow reactors, or spray column systems. It depends on the desired operating mode and scale. In laboratory setup, batch reactors are preferred due to their simplicity and ease of instrumentation. But for real-world applications, continuous or cyclic flow systems are more practical, as they mimic conditions in heat exchangers and storage loops. In these configurations, agitation has a critical role not only in enhancing gas-liquid contact but also in breaking agglomerates and maintaining a slurry-like consistency [84].

The solid content of a hydrate slurry ranges between 5% and 30% by volume. At lower hydrate fractions, the system behaves similarly to a Newtonian fluid with moderate viscosity. As the solid content increases, non-Newtonian characteristics such as yield stress and shear thinning become more significant and require precise control over operating parameters to avoid clogging and performance degradation [10].

Across different studies, the enhancement observed with nanoparticles appears to be dominated by heterogeneous nucleation effects rather than bulk thermal conductivity improvements alone. While increased thermal conductivity can help reduce local temperature gradients, experimental evidence consistently shows that induction time reduction correlates more strongly with the availability of solid nucleation sites and improved interfacial ordering. Significant variability remains across studies due to differences in particle surface chemistry, dispersion stability, and measurement protocols. From a design perspective, the most reliable benefit of nanoparticles lies in their ability to provide nucleation surfaces and improve formation repeatability rather than dramatically increasing heat transfer rates.

Among the mechanisms commonly proposed to explain thermal conductivity enhancement in nanofluids, only a subset is directly relevant to CO_2_ hydrate slurry systems. In hydrate-forming environments, the dominant contribution does not arise from classical nanoscale transport mechanisms alone, but from their interaction with phase change and multiphase flow processes. The most significant mechanism is the role of nanoparticles as heterogeneous nucleation sites, which reduces the free energy barrier for hydrate formation and leads to shorter induction times and more spatially distributed crystal growth. This behavior has been consistently observed in experimental studies involving graphene oxide, metal oxides, and hybrid nanofluids, where induction time reduction correlates more strongly with particle surface availability than with bulk thermal conductivity enhancement [74,75].

A second key mechanism is the enhancement of local heat dissipation at the hydrate–liquid interface. Because hydrate formation is strongly exothermic, localized heat accumulation reduces subcooling and suppresses further growth. Nanoparticles improve heat transport near the gas–liquid–solid interface, facilitating continuous crystallization and improving overall conversion efficiency [66,69]. In this context, thermal conductivity enhancement is coupled with improved heat removal rather than arising solely from intrinsic particle conductivity.

In contrast, mechanisms frequently cited in conventional nanofluid literature such as Brownian motion-induced microconvection and interfacial liquid layering are of secondary importance in hydrate slurry systems. The presence of solid hydrate particles and the dominance of macroscopic heat and mass transfer processes diminish the relative contribution of nanoscale transport effects [15,70]. Similarly, particle clustering and percolation networks, while capable of increasing effective thermal conductivity, are not necessarily beneficial in hydrate slurries, as they promote agglomeration, increase apparent viscosity, and can lead to flow instability or blockage under circulating conditions [76,77].

## 6. Thermal Conductivity Enhancement with Nanoparticles

CO_2_ hydrate slurries are attractive working media for cold thermal energy storage and gas separation, but their performance is constrained by slow formation kinetics and the difficulty of removing the heat released during crystal growth within a circulating solids-bearing fluid [85].

While conventional chemical promoters such as tetrahydrofuran, cyclopentane, tetrabutylammonium salts, light hydrocarbons, and surfactants like SDS or Tween 80 can accelerate formation process, their industrial use is tempered by volatility, environmental and toxicity concerns, and downstream separation burdens, motivating interest in solid-phase promoters like nanoparticles that can couple kinetic promotion with improved heat transport in the slurry phase [86,87].

Hydrate formation is exothermic, as crystals grow, local temperature rises reduce the thermodynamic driving force and can stall conversion, so the practical rate of CO_2_ hydrate slurry production is strongly tied to how fast the suspension can conduct and convect the released heat away from the formation front [85]. Nanoparticle dispersions (nanofluids) provide a route to raise the effective thermal conductivity of the continuous phase, with numerous measurements across Al_2_O_3_, Cu, CuO, SiO_2_, and carbonaceous particles showing conductivity gains that scale, within stability limits, with particle loading and often with temperature in the operating range relevant to hydrate systems [88,89].

In parallel, high-conductivity carbon frameworks show the fundamental potential of graphitic fillers to establish percolated heat paths, underscoring why carbon-based nanoparticles frequently outperform oxides at comparable volume fractions [90,91].

Despite the empirical gains, predicting the thermal behavior of nanoparticle-laden hydrates remains challenging because dispersion stability, pH, ionic strength, surfactant package, and shear history modulate both structure and transport at multiple scales [89].

Competing and possibly concurrent explanations like Brownian motion-assisted micro convection, interfacial liquid layering, percolation/clustering, ballistic phonon contributions across particle-liquid interfaces, and electrostatic effects are each supported to varying degrees depending on the chemistry and flow regime, and consensus on a single dominant mechanism has not emerged for hydrate slurries [15,89].

This mechanistic uncertainty complicates a priority nanoparticle selection and dose optimization for a given reactor or loop geometry. Within CO_2_ hydrate slurries specifically, nanoparticles can shorten induction times and increase gas uptake, yielding higher apparent formation rates and greater conversion under otherwise identical conditions [54].

Commonly used graphene oxide is a representative case: experiments at 279 K and 3–5 MPa reported induction time reductions of 53–74% and gas consumption increases of 5–16% relative to nanoparticle-free baselines, with performance sensitive to dose and pressure. Synergy with conventional kinetic promoters is common; combining graphene-based nanoparticles with SDS has repeatedly enhanced CO_2_ hydrate formation, indicating that surfactants aid both dispersion stability and interfacial mass transfer while the nanoparticles provide nucleation sites and heat-transfer pathways. The effect of nanoparticles must be evaluated together with slurry rheology because flowability determines pump work, exchanger coefficients, and the propensity for agglomeration in recirculating loops [92].

Recent in-loop studies of CO_2_ hydrate slurries report divergent behaviors from shear-thinning to apparent shear-thickening depending on solids fraction, particle/host chemistry, and measurement method, which makes generalization risky without system-specific testing.

Surfactants remain the primary handle on early-stage viscosity, but nanoparticle addition interacts with surfactant type and concentration, so combinations that accelerate kinetics can also either delay or hasten viscosity rise, with consequences for pressure drop and heat-exchange fouling in continuous service [10].

Beyond kinetics, nanoparticles have been shown to increase the effective thermal conductivity of hydrate-bearing systems themselves, complementing the enhancement of the carrier liquid and helping to evacuate formation heat from the crystal-liquid interface within the slurry microstructure [93].

Carbonaceous particles are particularly effective in this role due to their high intrinsic conductivity and tendency to form connected micro-networks, an effect consistent with broader nanofluid literature that ranks graphitic fillers among the strongest conductivity enhancers at low volume fractions [90,91]. As a result, improved thermal conductivity helps maintain more uniform temperature distribution within the reactor or flow loop during rapid hydrate formation. This supports stable growth and improves overall process efficiency [93].

Real-world implementation requires attention to dispersion stability because nanoparticles in cold, high-shear, saline environments can agglomerate and sediment, degrading both thermal properties and kinetics over time. Stabilization strategies include coupling nanoparticles with surfactants or polymeric dispersants and, in some systems, using ionic-liquid modifiers to maintain small, well-wetted aggregates under operating temperatures and shear rates typical of hydrate slurries. Effective dosages reported for hydrate promotion are modest tens of ppm to 0.1 wt% for many carbon and oxide nanoparticles with diminishing returns or outright inhibition when concentrations are pushed high enough to trigger clustering or mass-transfer blockage. Because these windows are narrow and system-specific, dose-response screening under the intended hydrodynamic and thermal conditions is an essential part of formulation. The advantages of nanoparticle-assisted CO_2_ hydrate slurries can be summarized as faster formation, higher gas uptake, and improved thermal management at comparatively low solid loadings, all of which support compact equipment and shorter cycle times [54].

The countervailing risks which need to be considered are increased viscosity and pressure drop, agglomeration and sedimentation under transients, and sensitivity to the exact pairing of particle chemistry with surfactant package and water chemistry, which together can erode the expected net gain in heat-transfer coefficients in long-duration operation. These trade-offs argue for integrated optimization by balancing kinetics, thermal transport, and rheology rather than pursuing any single metric in isolation [92].

The most promising route to practical deployment appears to be hybrid formulations that combine a low-dose, high-conductivity nanoparticle (often carbon-based) with a surfactant system tuned for dispersion stability and controlled agglomeration, validated in pilot-scale rigs that capture the shear histories and thermal shocks of real loops. Advances in understanding structure-property links particularly the roles of interfacial layering, clustering/percolation, and electrostatic interactions in cold, saline, multiphase flow should enable mechanistic selection of particle type, size, and surface chemistry rather than empirical trial-and-errors.These are coupled with loop-scale rheology and heat-transfer measurements, CO_2_ hydrate slurries formulated with nanoparticles have a credible further development and implementation path from laboratory demonstrations to robust, clean-energy thermal storage and separation processes [93].

In addition to oxides and carbon-based nanostructures, metallic nanoparticles such as copper (Cu), silver (Ag), and gold (Au) have attracted attention due to their superior intrinsic thermal conductivities (200–400 W·m^−1^·K^−1^) [94]. Dispersions of Cu nanoparticles in water have demonstrated up to 20–25% thermal conductivity enhancement at loads below 0.1 wt% [95], and Ag-based nanofluids have shown similar gains at even lower concentrations. These improvements arise from both the formation of conductive percolation networks and enhanced phonon transport across particle-fluid interfaces that aid in dissipating the exothermic heat of hydrate formation. Despite these advantages, metallic nanoparticles present challenges including oxidation, surface instability, and agglomeration under hydrate-forming conditions. Recent work shows hybrid formulations combining trace metallic nanoparticles with stabilizing oxides or carbon-based fillers can yield synergistic improvements in both thermal conductivity and dispersion stability by producing a balanced pathway for hydrate slurry applications [96]. Table 2 summarizes reported work on improving thermal conductivity in gas hydrate systems using nanoparticles and nanofluids. Different additives such as metal nanoparticles, carbon nanotubes, and metal oxides show clear increases in effective thermal conductivity across different operating conditions. This shows the potential of nanomaterials to improve heat transfer in hydrate systems.

Nanoparticles have been widely investigated as kinetic promoters for hydrate formation because they can simultaneously improve heterogeneous nucleation and enhance heat transfer within the hydrate-water system. However, the magnitude of these improvements varies significantly depending on nanoparticle type, concentration, and dispersion stability. Experimental studies report that the addition of nanoparticles such as graphene oxide (GO), graphite, CuO, or SiO_2_ typically increases the effective thermal conductivity of the base fluid by approximately 10–35% for concentrations between 0.01 and 0.1 wt%, primarily due to the high intrinsic conductivity of the particles and micro-scale convection induced by Brownian motion. This enhancement can improve heat removal during hydrate crystallization, which is particularly important because hydrate formation releases large amounts of heat that may otherwise inhibit further nucleation [99,100].

Beyond thermal conductivity effects, nanoparticles also act as heterogeneous nucleation sites that reduce the induction time for hydrate formation. For example, Yan et al. reported that graphene oxide nanoparticles reduced induction time by approximately 53–74% compared with pure water under conditions of 279 K and 3–5 MPa, while increasing CO_2_ gas consumption by 5–16% [54].

Similarly, experiments using CuO nanoparticles showed induction time reductions of roughly 30–50% depending on particle loading and mixing conditions. In some cases, hybrid promoter systems combining nanoparticles with surfactants have demonstrated even stronger effects [15].

Li et al. reported that a mixed promoter system consisting of 0.005 wt% GO and 0.2 wt% SDS shortened hydrate formation time by nearly 70% compared with pure water and increased gas uptake by approximately 11%. These results indicate that nanoparticle promotion is typically most effective within a relatively narrow concentration range. At higher concentrations, particle agglomeration or increased slurry viscosity may reduce gas-liquid mass transfer and offset the benefits of enhanced thermal conductivity [101].

## 7. Thermophysical Properties of CO_2_ Hydrate Slurries

The thermophysical behavior of CO_2_ hydrate slurries has an influence on their performance in cold thermal energy storage (CTES) and refrigeration systems. Among the key parameters, density, specific heat, and thermal conductivity determine both storage capacity and transport behavior. These properties are highly dependent on slurry composition, particularly the hydrate volume fraction, particle dispersion, and presence of additives. Understanding how these factors influence thermal performance is essential for the effective design and operation of hydrate-based systems.

### 7.1. Density and Specific Heat

Nanoparticles rarely change the intrinsic properties of the CO_2_ hydrate crystal; rather, they shift the slurry’s composition and microstructure, which are exactly what determine density and specific heat. The hydrate phase is denser and has a lower heat capacity than liquid water, so anything that increases the hydrate volume fraction, ϕ_h_, nudges the slurry toward higher density and lower sensible heat capacity. For CO_2_ hydrates, the phase density is 1.09–1.11 g·cm^−3^ near 0–5 °C, and many experimental and modeling papers now converge on a specific heat in the 2.1–2.7 kJ·kg^−1^·K^−1^ range both clearly different from water (4.18 kJ·kg^−1^·K^−1^) [102].

Now nanoparticles produce measurable improvement on density. At fixed temperature and pressure, the slurry density can be approximated by a volume-weighted mixture rule,

(2) $$\rho_{sl}=\left(1-\emptyset_{h}-\emptyset_{np}\right)\rho_{aq}+\emptyset_{h}\rho_{h}+\emptyset_{np}\rho_{np}$$

where, $\rho_{aq}$ is the carrier liquid (water or brine), $\rho_{h}$ the nanoparticle density. Because $\rho_{np}$ for common oxides and carbons (e.g., Al_2_O_3_, SiO_2_, graphite) exceeds $\rho_{aq}$ any non-zero nanoparticle volume fraction $\emptyset_{np}$ increases $\rho_{sl}$ slightly. In practice, however, $\emptyset_{np}$ is tiny (10–1000 ppm by mass in CTES work), so the direct density increase from the particles is marginal. The indirect effect dominates carbon-based nanoparticles (graphite, graphene oxide) and certain metal oxides consistently accelerate CO_2_-hydrate nucleation and growth, raising $\emptyset_{h}$ by increasing gas consumption at a given residence time. For example, graphite nanoparticles cut induction times by 80% and raised maximum CO_2_ uptake by 13% in Energy & Fuels experiments, while graphene-oxide promoters shortened induction by 53–74% and increased gas consumption by 5–16% in Energies both at sub 0.1 wt% loadings [103]. A higher $\emptyset_{h}$ pushes the mixture toward the hydrate’s 1.10 g·cm^−3^ density [104], which is why loop and rig studies report density climbing with hydrate formation.

### 7.2. Effective Thermal Conductivity of CO_2_ Hydrate Slurry

The thermal transport behavior of CO_2_ hydrate varies from that of crystalline ice, even though both consist of hydrogen-bonded water frameworks. In clathrate hydrates, guest molecules trapped within the water cages create vibrational degrees of freedom that strongly interact with lattice phonons. This produces resonant scattering, and reduces phonon mean-free paths giving rise to the nearly temperature-independent glass-like conductivity that characterizes hydrate structures. The low lattice conductivity is mainly due to energy exchange between localized guest vibrations and the host lattice acoustic modes, which suppresses collective phonon propagation through the framework. Early measurements of hydrate conductivity showed values much lower than those of ice. Experiments in the 265–280 K range reported conductivities between 0.45 and 0.70 W m^−1^ K^−1^, whereas ice Ih at 273 K has about 2.2 W m^−1^ K^−1^ [105].

Later tests on compact methane hydrate with approximately 90% occupancy and minimal porosity gave 0.68 ± 0.01 W m^−1^ K^−1^. It indicates that the porosity reduction only marginally increases thermal conductivity because the dominant heat-transfer limitation arises from molecular scattering rather than structural voids [106].

The measured thermal diffusivity of (2–3) × 10^−7^ m^2^ s^−1^ shows this correlation between conduction and stored heat capacity. Some computational studies show the origin of guest-dependent conductivity differences. Non-equilibrium molecular-dynamics simulations of structure-I hydrates found that methane hydrate has about 15–20% higher conductivity than CO_2_ and xenon hydrates across the 30–260 K range [107].

The heavier and larger CO_2_ molecules enhance lattice distortion and anharmonic scattering, shortening phonon relaxation times. Comparisons between empty, partially filled, and fully occupied frameworks revealed that empty hydrates behave similarly to ice Ih but with smaller absolute thermal conductivity value [108].

Pressure effects have been studied up to approximately 50 MPa, showing only a 10–15% increase in thermal conductivity with compression. The influence of external pressure is therefore secondary, since phonon scattering through guest-host coupling dominates. Within the typical formation range of 5–7 MPa used in laboratory and pilot systems, pressure has little measurable effect on the intrinsic heat-transfer coefficient of CO_2_ hydrate. Using transient hot-wire and plane-source techniques confirms the low and weakly temperature-dependent nature of hydrate conductivity. For semiclathrate hydrates such as TBAB and TBAC, values between 0.38 and 0.44 W m^−1^ K^−1^ were obtained in the 223–303 K interval with ±0.7% uncertainty. These values are roughly five times lower than ice and represent a close analogue to CO_2_ hydrate because both share similar cage dynamics. The nearly constant k with temperature shows that the hydrate network behaves as a strongly disordered medium for phonon transport, comparable to molecular glasses [109].

Thermal conductivity measurements of hydrate samples can be affected by residual unfrozen water near the probe, ice contamination, and microstructural heterogeneity such as micro-pores. If unfrozen water remains near the probe during measurement, it can produce an artificial jump near 273 K due to the four-fold difference between liquid and ice conductivities. Directional solidification during measurement forming hydrate from the bottom upward prevents such anomalies and ensures stable reading. The high reproducibility achieved under these conditions demonstrates the importance of microstructural control in obtaining intrinsic hydrate property data [110].

For system design, hydrate slurries are more relevant than static solids. CO_2_-hydrate slurry has dispersed solid hydrate particles in a carrier fluid (usually water, brine, or glycol). The effective thermal conductivity $k_{\mathrm{eff}}$ of such a two-phase mixture depends on the intrinsic conductivities of both phases, particle volume fraction (ϕ), particle morphology, and the interfacial thermal resistance between solid and liquid phases. Because both CO_2_ hydrate and water have comparable k values around 0.5–0.6 W m^−1^ K^−1^, the contrast is small. Analytical estimates using Maxwell-Eucken theory predict that increasing the solid fraction up to 30% only raises $k_{\mathrm{eff}}$ marginally to about 0.55–0.57 W m^−1^ K^−1^. Flow-cell experiments on CO_2_ and mixed-gas hydrate slurries confirm that thermal resistance is concentrated at the particle-liquid interface and within the dispersed layer rather than in the solid itself. The limiting factor in heat transfer during hydrate growth and melting is thus the interfacial contact conductance. As solid loading increases, conduction paths remain discontinuous, and convective renewal dominates over lattice conduction. Even at high hydrate fractions, $k_{\mathrm{eff}}$ stays close to the carrier fluid value unless firm mechanical compression is used to reduce interfacial voids [111].

Molecular-scale analyses of CO_2_ hydrate under partial occupancy or external electric fields reveal an even lower K (0.3 W m^−1^ K^−1^), since distortion of the lattice further enhances scattering and shortens energy-correlation times of vibrational modes. This result supports the interpretation that CO_2_ hydrate behaves like an amorphous solid with respect to heat transport [112].

In hydrate-bearing sediments, the same physical principles apply macroscopically. When hydrate replaces pore water, overall conductivity decreases because k_hydrate_ < k_quartz_. Laboratory studies on synthetic CO_2_/CH_4_ hydrates in sand report effective values of 1.2–1.6 W m^−1^ K^−1^ at 50% saturation, which are lower than equivalent water-saturated sediments. Thus, hydrate formation in geological formations acts as a thermal barrier by restricting heat flow during gas exchange or dissociation processes [113].

### 7.3. Rheological Properties and Flow Behavior

Because of high energy density, CO_2_ hydrate slurries are considered for thermofluid and secondary refrigerant applications. The flow behavior of CO_2_ hydrate slurries is governed by the way solid hydrate particles form, collide, and reorganize within the surrounding liquid. As hydrates grow, the slurry gradually shifts from a simple liquid to a complex suspension whose resistance to flow depends on both particle concentration and the forces acting between particles. For this reason, the rheology of hydrate slurries cannot be reduced to a single viscosity value; instead, it evolves continuously with formation progress, operating conditions, and shear history [114].

A wide range of experimental systems has been used to characterize CO_2_ hydrate slurry rheology, and these setups collectively show how strongly measurement conditions influence the interpretation of flow behavior. Early laboratory configurations relied on stainless-steel flow loops combined with capillary-based viscosity estimation. These small-volume systems with internal diameters of 7–10 mm provide good source to track hydrate formation and quantify pressure-drop responses as the solid phase grew. Their results consistently showed that even a small increase in hydrate content leads to rapid increases in resistance to flow [115].

Later flow-loop studies expanded the range of hydrate volume fractions tested and demonstrated how quickly a suspension transitions from a freely flowing liquid to a structured and increasingly resistant slurry. These experiments generally operated between 2.3 and 3.0 MPa and temperatures near 275–278 K, revealing clear shear-dependent behavior once the hydrate fraction reached roughly 10–15 vol% [116].

More advanced systems incorporated both dynamic flow-loops and mechanically agitated tanks. These arrangements allowed the slurry to be exposed to controlled shear while simultaneously monitoring pressure drops and flow stability. Across hydrate contents ranging from dilute to highly concentrated, the data showed that shear thinning becomes progressively stronger as the solid phase builds up, indicating the formation and subsequent breakup of particle networks under shear [16].

**Figure 6:**
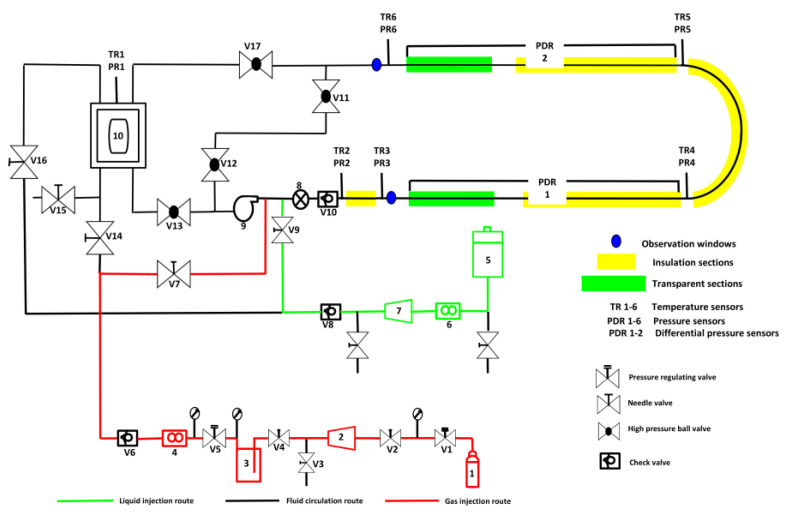
Schematic Diagram of the Instrumented Gas Hydrate Slurry Flow Loop with Liquid and Gas Injection Routes. (1) Gas cylinder; (2) gas booster pump; (3) buffer tank; (4) gas mass flow meter; (5) liquid storage tank; (6) turbine flowmeter; (7) liquid booster pump; (8) mass flowmeter; (9) circulating pump; and (10) high-pressure reactor. Adapted with permission from Lv et al., *ACS Omega* **2022**, *7*, 2444–2457. Copyright © 2022 American Chemical Society [117].

Figure 6 shows the high-pressure flow loops introduced in later work extended slurry studies into multiphase liquid environments, including cases where hydrates formed simultaneously in water and hydrocarbon phases. These systems highlighted that slurry rheology is not governed solely by solid fraction but also by interfacial behavior between immiscible phases. Under pressures near 3 MPa, mixed liquid environments exhibited distinct aggregation patterns and greater sensitivity to shear history [117].

Chemical additives form another major factor governing slurry flowability. Surfactants such as Caflon, Tween 80, OP-10, SDS, and similar compounds reduce particle-particle attraction by imposing steric or electrostatic repulsion. Flow-loop and rheometer tests at pressures of 2.3–3.5 MPa show that these additives suppress agglomeration and significantly lower apparent viscosity, even when hydrate fraction remains unchanged [48].

The interfacial tension (IFT) between CO_2_ hydrate particles and the surrounding liquid phase plays a decisive role in how hydrate slurries behave during flow. Low IFT values promote particle-particle attraction, making the solids more likely to cluster and form dense agglomerates. This aggregation strongly influences viscosity, yield stress, and overall flow stability. It directly affects whether a slurry remains mobile or transitions into a more structured network. Measurements of CO_2_ hydrate IFT in the literature shows considerable variation. Values reported range from approximately 1–4 mN/m in some systems to above 30 mN/m in others. These discrepancies came from differences in measurement techniques, system composition, pressure-temperature conditions, and whether additives such as TBAC, APG, or SDS were present. As a result, IFT must be interpreted in the context of the experimental setup rather than as a universal material property.

To provide a clearer comparison across systems, the range of reported IFT values is summarized in Table 3. This compiles measurements from water, brine, and additive-containing systems under different thermodynamic conditions.

### 7.4. Viscosity Models and Slurry Flow Behavior

Several empirical correlations have been proposed to describe the apparent viscosity of CO_2_ hydrate slurries flowing in pipelines under laminar conditions. These models are derived primarily from capillary viscometer measurements in closed flow loops and relate the apparent viscosity to the hydrate volume fraction and shear rate. Although the mathematical forms are different, all the models are rooted in experimental pressure-drop data and show the evolving microstructure of hydrate particles suspended in an aqueous phase.

For CO_2_ hydrate slurries formed in pure water, the apparent viscosity has been expressed as a strong function of hydrate volume fraction and shear rate. The proposed correlation takes the form [116]:

(3) $$\mu_{\mathrm{app}}=1900\left(\frac{2\phi_{s}^{3.6}}{{\dot{\gamma }}_{w}}+\phi_{s}^{5.4}{\dot{\gamma }}_{w}^{-1-0.77(1+ln\;\phi_{s})}\right)$$

This formulation captures the rapid increase in viscosity even at moderate hydrate loadings. The first term represents the contribution of dispersed hydrate particles at low shear rates, while the second term accounts for shear-dependent restructuring of hydrate aggregates. The strong dependence on $\phi_{s}$ reflects the onset of particle-particle interactions and early agglomeration observed experimentally in hydrate-water slurries

A simplified exponential expression has also been proposed for CO_2_ hydrate slurries in water [16],

(4) $$\mu_{\mathrm{app}}=0.0018exp\left(17.976\;\phi_{s}\right){\dot{\gamma }}_{w}^{-1.82\phi_{s}}$$

This model emphasizes the exponential sensitivity of viscosity to hydrate concentration while retaining shear-rate dependence. The decreasing exponent on shear rate indicates shear-thinning behavior at higher hydrate fractions, consistent with particle alignment and breakup of loose agglomerates under increasing flow intensity

When tetra-n-butyl phosphonium bromide (TBPB) is present, the apparent viscosity follows a different trend [45]:

(5) $$\mu_{\mathrm{app}}=5.0\times 10^{-4}exp\left(52.78\phi_{s}\right){\dot{\gamma }}_{w}^{1.387\exp\left(-10.307\phi_{s}\right)}$$

In this case, the model reflects the combined influence of hydrate fraction and the stabilizing effect of TBPB on particle dispersion. The positive exponent on shear rate indicates shear-thickening behavior, attributed to the formation of more compact hydrate clusters under flow. This behavior contrasts with hydrate-water systems and highlights the strong role of additives in modifying slurry rheology.

For CO_2_ hydrate slurries formed in the presence of sodium dodecyl sulfate (SDS), the apparent viscosity is given by [18]:

(6) $$\mu_{\mathrm{app}}=0.0125exp\left(18.65\phi_{s}^{1.315}\right){\dot{\gamma }}_{w}^{-1-1.077\phi_{s}+0.931}$$

This correlation shows a reduced sensitivity of viscosity to hydrate fractions compared with additive-free systems. The negative shear-rate exponent confirms shear-thinning behavior over the investigated range. The reduced viscosity increase at high hydrate fractions is consistent with the anti-agglomeration effect of SDS, which limits the formation of large hydrate clusters and improves slurry flowability

For hydrate slurries stabilized using Span-80, the apparent viscosity is expressed as [117]:

(7) $$\mu_{\mathrm{app}}=exp\left(-4.7798+0.2777\phi_{s}+24.3751\phi_{s}^{2}\right){\dot{\gamma }}_{w}^{-0.4352\phi_{s}-3.2395\phi_{s}^{2}}$$

This polynomial-exponential structure reflects the complex balance between particle stabilization and increasing solid content. At low hydrate fractions, viscosity remains relatively moderate, while higher fractions lead to pronounced non-Newtonian behavior. The formulation highlights the nonlinear role of surfactant concentration on hydrate particle interactions and flow resistance.

To address a broad range of hydrate concentrations, a piecewise formulation has been proposed [92]:

(8) $$\mathrm{For}\;\phi_{s}<8.6\%:\;\mu_{\mathrm{app}}=0.0034\;\phi_{s}^{0.1763}{\dot{\gamma }}_{w}^{1.9239\phi_{s}^{0.048}-1}$$

(9) $$\mathrm{For}\;\phi_{s}\geq 8.6\%:\;\mu_{\mathrm{app}}=0.0144\;\phi_{s}^{0.4381}{\dot{\gamma }}_{w}^{2.0753\phi_{s}^{0.1224}-1}$$

This formulation distinguishes two rheological regimes. Below the transition concentration, hydrate particles remain relatively dispersed, leading to weak shear dependence. Beyond this threshold, stronger particle networks develop and produce higher apparent viscosities and more pronounced non-Newtonian effects. The transition reflects a structural change in the slurry rather than a purely hydrodynamic effect. The most direct contribution to increased viscosity and yield stress in hydrate slurries is the hydrate volume fraction, typically ranging from 5 to 30 vol% in practical systems. At lower concentrations (<10 vol%), hydrate particles are dispersed sufficiently to allow free movement of the liquid phase around them, leading to modest increases in effective viscosity and Newtonian-like flow behavior. However, as the hydrate fraction increases, interparticle spacing diminishes, enhancing hydrodynamic interactions and promoting short-range ordering or clustering effects.

Sahu et al. conducted high-pressure rheological experiments and observed that increasing the hydrate volume fraction from 10% to 30% led to a nonlinear, near-exponential increase in effective viscosity, nearly quadrupling at a constant shear rate. This behavior arises due to steric hindrance, increased excluded volume effects, and the formation of percolating particle networks that resist deformation. These observations align with colloidal suspension theory, where critical packing thresholds mark the transition to yield stress and non-Newtonian shear-thinning flow. In addition to impacting viscosity, hydrate loading also determines whether the slurry exhibits plugging tendencies, wall slip, or shear banding, particularly in narrow channel geometries. Therefore, careful control of hydrate formation kinetics and fraction is essential for ensuring stable and predictable flow, particularly in continuous systems such as pipelines and looped cooling networks. The size, shape, and surface properties of hydrate particles significantly influence both the static and dynamic rheology of the slurry. Fine, and spherical particles promote lower viscosity due to better fluid accommodation and reduced drag. In comparison, irregular or large agglomerated particles increase the hydrodynamic radius and disrupt flow symmetry and this leads to higher energy dissipation under shear [10].

The presence of surfactants such as SDS can reduce particle agglomeration by imparting electrostatic repulsion, thereby maintaining smaller effective particle diameters and improving dispersion. However, such additives may also alter interfacial tension and modify lubrication layers between particles, introducing a secondary influence on rheological characteristics [124]. CO_2_ hydrate slurry is a multiphase flow system where solid hydrate particles form directly inside an aqueous phase under elevated pressure and low temperature. The solid phase is generated in situ through gas-liquid phase transformation and remains thermodynamically active during transport. Hydrate particles that are grown dissociate and evolve as local pressure temperature and shear conditions change. This dynamic behavior produces continuous variation in particle size surface roughness and interparticle bonding. Capillary viscometer experiments on CO_2_ hydrate slurry showed time-dependent viscosity even at fixed hydrate fraction confirming that hydrate particles do not behave as inert solids as where hydrate fractions between 4 vol% and 20 vol% produced apparent viscosities ranging from 3.8 mPa·s to 42.2 mPa·s under shear rates between 500 s^−1^ and 1000 s^−1^.

Table 4 summarizes representative experimental observations of hydrate slurry rheology obtained using capillary-based viscometry under laminar flow conditions. The data demonstrate that hydrate slurry flow behavior is strongly dependent on hydrate fraction, applied shear rate, and the presence of additives. For CO_2_ hydrate slurries, increasing hydrate fraction leads to clear rheological regime transitions, progressing from dilatant behavior at low hydrate loadings to Herschel-Bulkley and Bingham plastic behavior as particle interactions and network formation intensify. The inclusion of additives stabilizes hydrate particles and suppresses non-Newtonian effects, yielding near-Newtonian flow at comparable hydrate fractions. Shear-rate-dependent transitions observed in methane hydrate systems show that hydrate slurry viscosity cannot be described by a single constitutive model, and the concentration and shear-dependent viscosity correlations for predicting slurry flow performance is needed [92].

## 8. Technology Bottlenecks Which Still Need to Be Solved

Commercialization of CO_2_ hydrates, which can be crucial for carbon capture and thermal energy storage applications, faces significant challenges in terms of kinetic and thermodynamic limitations. In addition to the type of reactor, the CO_2_ hydrate formation is highly dependent on factors such as temperature, pressure, and the presence of promoters or inhibitors. Despite the substantial progress made in understanding the phase behavior and thermodynamics of CO_2_ hydrates, the slow kinetics of hydrate formation at lower pressures and temperatures remain a bottleneck. This results in long induction times and low overall conversion rates, which hinder the efficiency of CO_2_ hydrate-based systems. Researchers are investigating new gas mixes with CO_2_, new nucleation promoters, such as nanoparticles, amino acids, and surfactants, to enhance the rate of formation and reduce induction time. However, the scalability of these methods and their cost-effectiveness for large-scale applications remain unresolved.

The long-term stability of CO_2_ hydrates is another significant challenge. Hydrates tend to dissociate when subjected to changes in pressure or temperature, especially over extended periods. In practical applications like thermal energy storage and CO_2_ sequestration, maintaining stable hydrate formation for long durations is essential to ensure that the stored CO_2_ does not escape into the atmosphere. Research is focused on identifying methods to stabilize hydrates, including the use of tailored promoters, additives, and novel reactor designs. For instance, the incorporation of solid particles such as silica and carbon nanotubes into CO_2_ hydrate slurries has shown promise in increasing the stability of the hydrate structure. However, the trade-off between improved stability and the potential for increased costs due to these additives presents a significant barrier for widespread adoption. Scalability is one of the most pressing issues in the commercial implementation of CO_2_ hydrate-based technologies. Lab-scale reactors have demonstrated the potential of CO_2_ hydrate formation for energy storage and CO_2_ capture, but scaling up these systems to industrial levels remains problematic. Challenges include managing large volumes of CO_2_ and water under high-pressure conditions, maintaining efficient heat and mass transfer within the reactor, and ensuring uniform flow dynamics to promote efficient hydrate formation. The integration of microchannel reactors has been proposed as a solution to enhance heat transfer and reduce the size of the reactors, but there are still significant challenges in maintaining the stability of CO_2_ hydrates in microchannel systems. The complexity and cost of such systems can limit their commercial viability, requiring innovations in both reactor design and process control strategies.

Transporting and handling CO_2_ hydrate slurries introduces another set of challenges, particularly related to the slurry’s rheological behavior. Hydrate slurries exhibit non-Newtonian flow behavior, with shear-thinning or shear-thickening characteristics that depend on the hydrate concentration and shear rate. This behavior complicates the design of pumping systems and piping infrastructure for large-scale applications, as efficient and stable slurry transport is required for continuous operations. The potential for hydrate agglomeration and blockages in pipelines poses a significant risk for system performance. To address these issues, researchers are investigating the role of nanoparticles and surfactants in stabilizing hydrate particles and preventing aggregation, but the practical implementation of these solutions at a commercial scale is still uncertain.

The environmental and economic feasibility of CO_2_ hydrate-based technologies remains a key concern. While CO_2_ hydrate systems offer a promising method for carbon sequestration and thermal energy storage, their economic viability depends on the cost of implementing the necessary infrastructure, including high-pressure reactors, slurry pumps, and heat exchangers. The operational costs, particularly the energy required to maintain the high-pressure and low-temperature conditions needed for hydrate formation, are significant barriers to their widespread adoption. Environmental concerns related to the long-term storage of CO_2_ in hydrates, including the potential risks of leakage or dissociation, need to be carefully addressed through rigorous monitoring and regulatory frameworks. The development of cost-effective and environmentally sustainable technologies to capture, store, and utilize CO_2_ hydrates will require continued investment in both research and infrastructure.

## 9. Conclusions

This review focused on CO_2_ hydrate slurries from a thermofluid-based point of view, with attention to how they form, how they flow, and how they transfer heat in practical applications. CO_2_ hydrate slurries store cold through phase change while still behaving as a pumpable fluid. Compared with bulk hydrate beds, slurry systems allow heat to be extracted continuously. The dissociation enthalpy of CO_2_ hydrates that is reported in the range of about 350 to 520 kJ per kilogram of water and is higher than that of ice. This energy can be delivered near freezing temperatures, which makes these systems well suited for cooling and HVAC applications. Hydrate formation remains one of the main constraints affecting future commercial applications. Nucleation is slow and irregular in many systems, and heat released during formation can raise local temperature and slow further growth. Slurry-based configurations help reduce these limitations by keeping hydrate particles in motion and continuously renewing the gas-liquid interface. Experimental studies show that hydrate fraction, particle size, and shear rate strongly influence formation rate and stability, and these factors must be controlled together rather than treated independently.

A wide range of catalysts and additives have been studied to improve hydrate formation and maintain slurry stability. Thermodynamic promoters such as tetrahydrofuran and cyclopentane help shift hydrate formation to milder operating conditions and while surfactants like SDS improve gas-liquid contact and reduce induction time. Highly conductive nanoparticles such as metal oxides and carbon-based materials can provide additional nucleation sites and help remove heat release during formation and this supports faster growth. More recently, amino acids have also been explored as environmentally friendly promoters with promising results. Choosing the right additive depends on balancing performance improvement, environmental impact, and long-term slurry stability. Reactor design is also important for achieving stable and continuous hydrate slurry production. Effective mixing and efficient heat removal helps maintain uniform temperature and prevent particle agglomeration or blockage during operation. Continuous stirred tank reactors and flow-loop systems are commonly used because they provide better control over operating conditions and allow longer steady operation. Proper reactor geometry, impeller selection, and residence time distribution are critical for maintaining uniform hydrate particle distribution and preventing localized overheating during formation. The flow behavior of CO_2_ hydrate slurries changes as solid content increases. At low hydrate fractions, the slurry behaves close to a Newtonian fluid and can be circulated using conventional pumps. As the hydrate fraction rises, particle interactions increase flow resistance and lead to shear-dependent behavior. Beyond a certain solid loading, pressure drops increase rapidly and flow stability decreases. For most systems, stable operation requires hydrate fractions below roughly 20 to 25 percent volume percent.

Heat transfer in CO_2_ hydrate slurries is limited by the low thermal conductivity of the hydrate phase itself. Unlike ice, hydrate crystals do not conduct heat efficiently, so heat transfer depends strongly on particle motion and contact with heat transfer surfaces. Slurry flow improves this process by constantly renewing the interface, while compact heat exchangers and microchannel geometries further enhance heat removal by increasing surface area and shear. These features lead to faster thermal response compared with bulk hydrate systems. Nanoparticles have shown potential to improve hydrate slurry performance when used at low concentrations. They reduce induction time and increase gas uptake by providing additional nucleation sites, and they can increase the effective thermal conductivity of the carrier liquid. Carbon-based and metal-oxide nanoparticles show benefits at concentrations below about 0.1 wt%, while higher concentrations may increase viscosity and lead to stability issues. The balance between thermal improvement and flow resistance is therefore critical.

Several challenges remain before CO_2_ hydrate slurries can be deployed at large scale. Continuous production under steady operating conditions is still difficult due to the narrow thermodynamic window required for hydrate stability. Long-term slurry stability, pressure fluctuations, and mechanical wear also require further investigation. Addressing these issues will require improved reactor designs with better mixing and heat removal, more robust process monitoring and control strategies, and careful material selection to reduce equipment degradation. Existing thermodynamic and hydrodynamic models still have limited capability in predicting slurry behavior under real flow and heat transfer conditions. Future work should focus on integrating high-quality experimental data with multiphase CFD and rheological modeling to develop validated predictive frameworks that can accurately capture particle interactions, heat transfer behavior, and flow stability across different operating regimes.

## Figures and Tables

**Table 1:** Various thermodynamic promoters and their effects on phase equilibrium curves for CO_2_.

Thermodynamic Hydrate Promoter	Promoter Temperature Range (K)	Promoter Pressure Range (MPa)	Promoter Concentration	References
Tetrahydrofuran (THF)	274.5–287.7	0.11–3.286	1.4–25.03 wt%	[70,71]
Tetrabutylammonium bromide (TBAB)	279.40–291.75	0.34–4.55	4.43–60 wt%	[72,73]
Cyclopentane (CP)	279.26–292.4	0.55–3.64	2–7.3 wt%	[71,73]
Propane (C_3_H_8_)	274–282	0.5–3.7	3–60 mol% (in CO_2_ + C_3_H_8_ feed gas)	[74]
Methane (CH_4_)	273–289	3.1–6.8 (composition-dependent; broader reports span 1.5–7)	10–90 mol% (in CO_2_ + CH_4_ feed gas)	[75,76]

**Table 2:** Summary of Reported Thermal Conductivity Enhancement in Gas Hydrate Systems Using Nanoparticles and Nanofluids.

System	Nanoparticle Type	Thermal Conductivity (k) (W·m^−1^·K^−1^)	Temperature/Pressure	Reference
THF Hydrate + Cu Nanoparticles	Cu NPs, 0.1–1 wt %	0.55–0.64 (W·m^−1^·K^−1^), 16% vs. pure THF hydrate	263 K, 1 atm	[97]
THF Hydrate + CNT (MWCNT)	CNT 0.1–1 wt %	0.55–0.68 (W·m^−1^·K^−1^), 24%	260 K (−13 °C), 1 atm	[15]
CO_2_ Hydrate Formation in Water + Al_2_O_3_ Nanofluid	Al_2_O_3_ NPs (1 wt %) + SDS surfactant	Effective (k)–0.72 vs. 0.58 (pure water hydrate)–24%	273 K, 3.5 MPa CO_2_	[54]
Methane Hydrate Formation + Graphene Oxide (GO)	GO (0.02 wt %) + SDS	Effective (k)–0.75 (W·m^−1^·K^−1^) vs. 0.6 (W·m^−1^·K^−1^) baseline	273 K, 8 MPa CH_4_	[98]

**Table 3:** Reported interfacial tension (IFT) ranges for gas hydrate-water systems with and without additives under representative temperature and pressure conditions.

System Type	IFT Range (mN/m)	Typical T (K)	Typical P (MPa)	References
Hydrate + Water (pure system)	1.7–21	266–285	2–6	[118,119]
Hydrate + Water + TBAC/TBPB/Salts	3.8–4.7	275	2.5	[120]
Hydrate + Water + APG/Surfactant blends	3.8–4.0	275–277	2.5	[120]
Hydrate + Water + SDS (100–500 ppm)	26–39	279	3.5	[121]
Hydrate + Water at High Pressure (40 MPa)	29–30	287	40	[122,123]

**Table 4:** Summary of Rheological Behavior and Apparent Viscosity of CO_2_ Hydrate Slurries under Different Hydrate Fractions and Shear Rates.

Hydrate Slurry Type	Experimental Method	Hydrate Fraction (vol%)	Rheological Behavior	Apparent Viscosity (mPa·s)	Shear Rate (s^−1^)	References
CO_2_ hydrate	Ostwald viscometer (Di = 16 mm)	4–20	αₕydr < 5 vol%: Dilatant 5 ≤ αₕydr < 10 vol%: HB-type (dilatant trend) αₕydr ≈ 10 vol%: Bingham plastic αₕydr > 10 vol%: HB-type (pseudoplastic trend)	3.8–42.2	500–1000	[116]
CO_2_ hydrate with additives	Ostwald viscometer (Di = 5 mm)	4–10	4 ≤ αₕydr < 10 vol%: Newtonian (Caflon)	7–15 (at 600 s^−1^)	500–750	[48]
TBPB hydrate	Ostwald viscometer (Di = 8 mm)	0–28.2	Pseudoplastic	4–41	130–700	[124]
CH_4_ hydrate	Ostwald viscometer (Di = 25.4 mm)	6–11	$20\;\leq \dot{\gamma }$ ≤ 180 s^−1^$:\;\mathrm{Pseudoplastic}\;180\;<\;\dot{\gamma }$ < 300 s^−1^$:\;\mathrm{HB}-\mathrm{type}\;(\mathrm{transition})\;\dot{\gamma }$ ≥ 300 s^−1^: Dilatant	3–50	20–700	[125]
CO_2_ hydrate	Ostwald viscometer (Di = 50 mm)	1.4–17.2	Dilatant	12–520	40–590	[92]

## Data Availability

No new data were created or analyzed in this study. Data sharing is not applicable to this article.
